# The Genus *Alnus*, A Comprehensive Outline of Its Chemical Constituents and Biological Activities

**DOI:** 10.3390/molecules22081383

**Published:** 2017-08-21

**Authors:** Xueyang Ren, Ting He, Yanli Chang, Yicheng Zhao, Xiaoyi Chen, Shaojuan Bai, Le Wang, Meng Shen, Gaimei She

**Affiliations:** School of Chinese Pharmacy, Beijing University of Chinese Medicine, Beijing 100102, China; renxueyang1996@163.com (X.R.); heting0572@126.com (T.H.); changyanli94@163.com (Y.C.); zhaoyicheng0824@163.com (Y.Z.); chenxiaofly1209@126.com (X.C.); shaojuanbai@163.com (S.B.); wangle17@126.com (L.W.); shenmeng44@163.com (M.S.)

**Keywords:** chemical constituents, biological activities, *Alnus*, diarylheptanoids

## Abstract

The genus *Alnus* (Betulaceae) is comprised of more than 40 species. Many species of this genus have a long history of use in folk medicines. Phytochemical investigations have revealed the presence of diarylheptanoids, polyphenols, flavonoids, terpenoids, steroids and other compounds. Diarylheptanoids, natural products with a 1,7-diphenylheptane structural skeleton, are the dominant constituents in the genus, whose anticancer effect has been brought into focus. Pure compounds and crude extracts from the genus exhibit a wide spectrum of pharmacological activities both in vitro and in vivo. This paper compiles 273 naturally occurring compounds from the genus *Alnus* along with their structures and pharmacological activities, as reported in 138 references.

## 1. Introduction

*Alnus* is a genus in the family Betulaceae, which comprises more than 40 species mainly distributed in Asia, Africa, Europe and North America. A total of seven species and one variant are distributed in the south and north of China [1]. The plants in the genus are commonly used as traditional medicines [2]. *Alnus hirsuta* Turcz. indigenously distributed in Korea, China, Japan, and Russia, has been used in Oriental medicine as a remedy for fever, haemorrhages, burn injuries, diarrhea, and alcoholism [3]. It was reported that many bioactive natural components including diarylheptanoids, polyphenols, flavonoids, terpenoids, and steroids, were isolated from the genus [4,5,6,7,8]. Diarylheptanoids comprise a class of natural products formed from 1,7-diphenylheptane, which appears in linear and cyclic forms [9]. They have been regarded as the primary bioactive compounds of *Alnus* and have drawn attention due to their physiological activities, especially their anticancer and antioxidative activities [10,11].

*Alnus japonica* Steudel., *Alnus hirsuta* Turcz. and *Alnus glutinosa* (L.) Gaertn. have a long-standing medical history and extensive research on their irreplaceable functions has been reported. *A*. *japonica*, a famous medical herb in China, Japan and Korea as well as a functional food consumed in health drinks, contains abundant diarylheptanoid derivatives, in addition to methylated and acylated diarylheptanoids and diarylheptanoid glycosides [12,13,14]. It has been known to exert antioxidative, anti-inflammatory, anticancer and hepatoprotective effects and its antioxidative properties are presumed to contribute to its hepatoprotective activity in certain situations [15]. Diarylheptanoids with strong antioxidative activity isolated from *A*. *japonica* and *A*. *hirsuta* showed significant hepatoprotective effects on *t*-butyl hydroperoxide (*t*-BHP)-induced toxicity in primary rat hepatocytes and a human hepatoma cell line (HepG2), respectively [3,16]. *Alnus glutinosa* (L.) Gaertn., used on the market as a food supplement to help reduce the risk of different chronic dermatological conditions, has increasingly become a research hot-spot for its potent chemo-protective, antioxidant and antimicrobial effects [17,18,19]. Diarylheptanoids from the bark of *A*. *glutinosa* may serve as protectors of normal cells during chemotherapy without significantly diminishing the effect of the applied chemotherapeutic agents [4,20].

Sati et al. have summarized 192 chemical constituents and biological activities of genus *Alnus* [2]. Further studies on the *Alnus* genus were carried out in recent years, and many chemical components from this genus have been isolated. Therefore, a new comprehensive and systematic review of *Alnus* genus is much needed. Most of the papers not only covered new chemical components, but also refered to their pharmacology activities, structure-activity relationships and functional mechanisms, especially the anticancer meshanism of hirsutenone [11,21,22,23]. Hirsutenone can sensitize resistant ovarian cancer cells to cisplatin, so that co-treatment with it may have the potential to overcome chemoresistance [24]. Futhermore, an induction of oxidative stress and topo II-mediated DNA damage may play a role in hirsutanone-induced cancer cell death [21]. In this review, we mainly summarized 273 chemical constituents and biological activities of the genus *Alnus*, based on 138 cited references. It is hoped that the information presented in this paper will be useful for further research and the application of this genus.

## 2. Chemical Constituents

**Figure 1:**
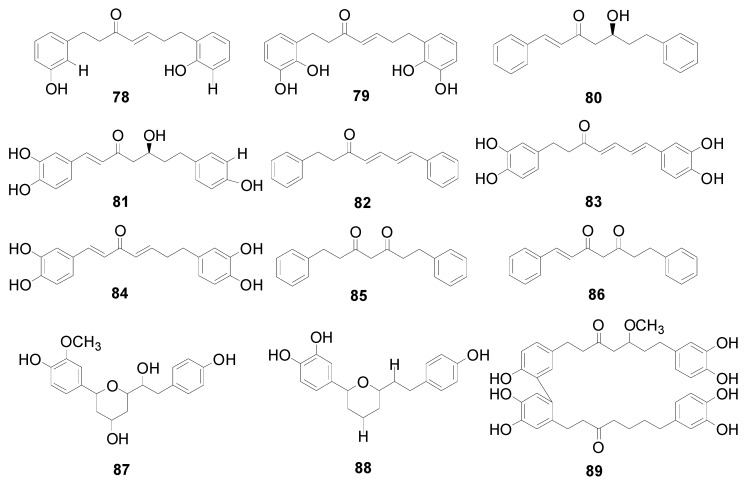
Structures of compounds **78**–**89**.

**Figure 2:**
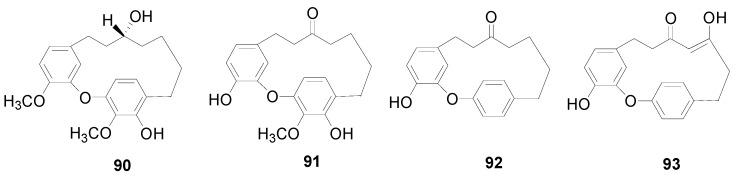
Structures of compounds **90**–**93**.

**Figure 3:**
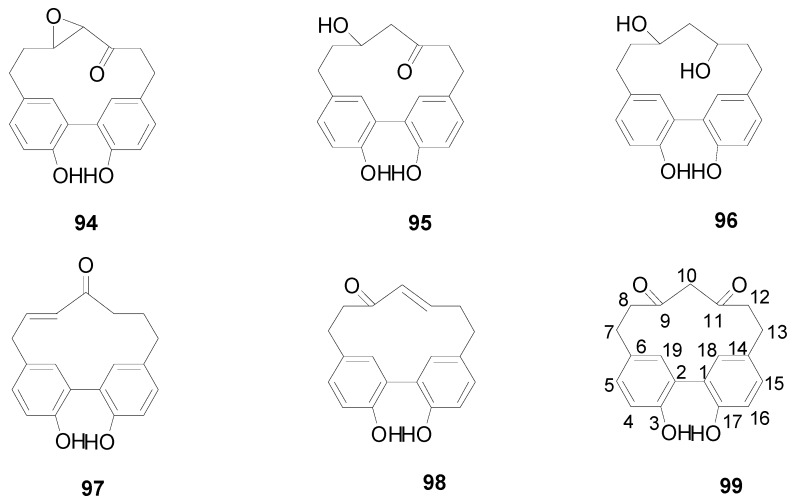
Structures of compounds **94**–**99**.

**Figure 4:**
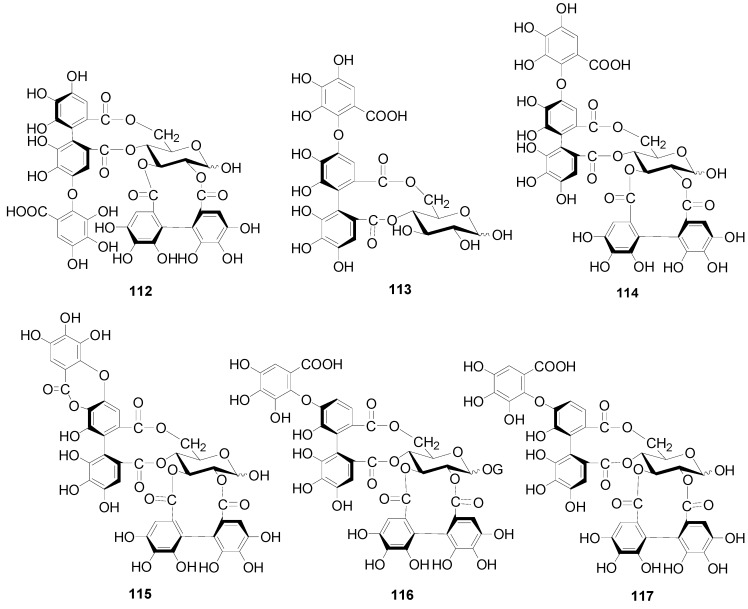
Structures of compounds **112**–**117**.

**Figure 5:**
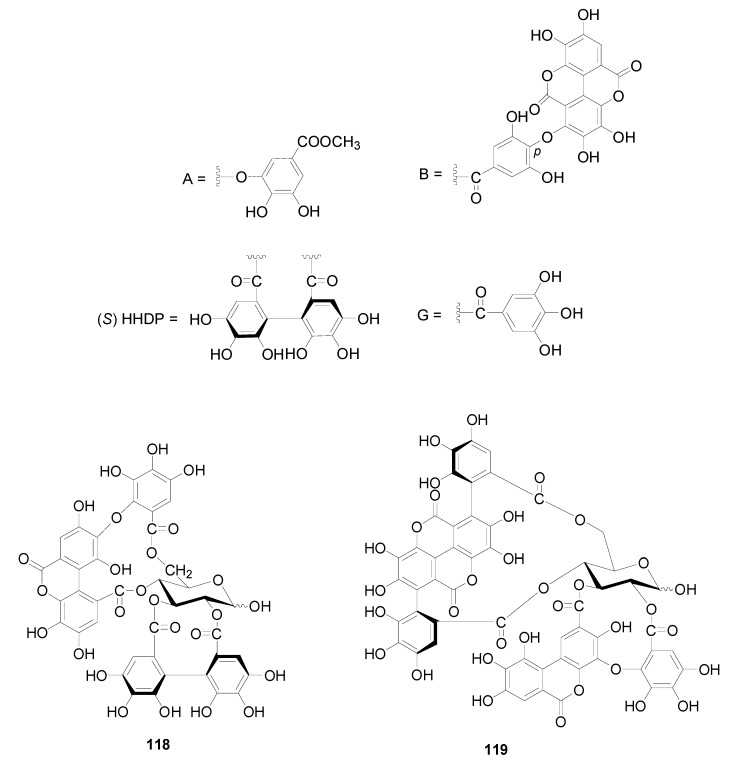
Structures of compounds **118** and **119**.

**Figure 6:**
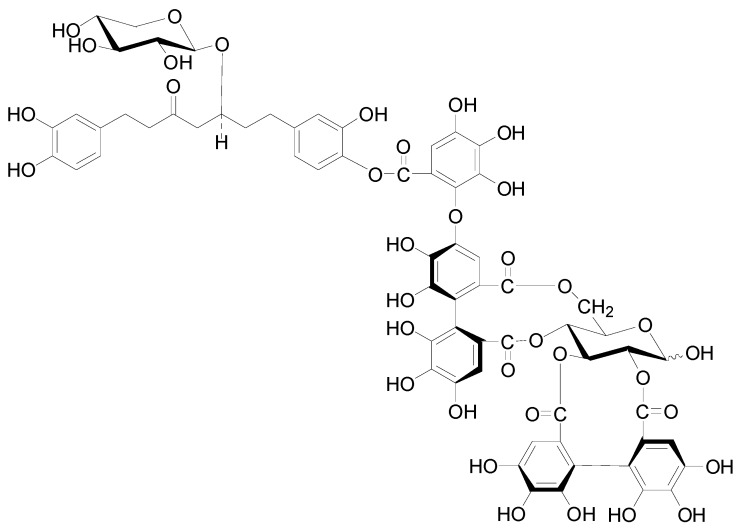
Structures of compounds **120**.

**Figure 7:**
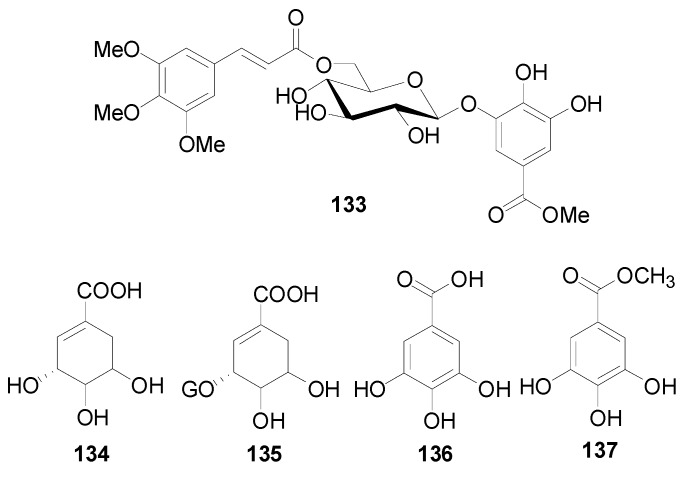
Structures of compounds **133**–**137**.

**Figure 8:**
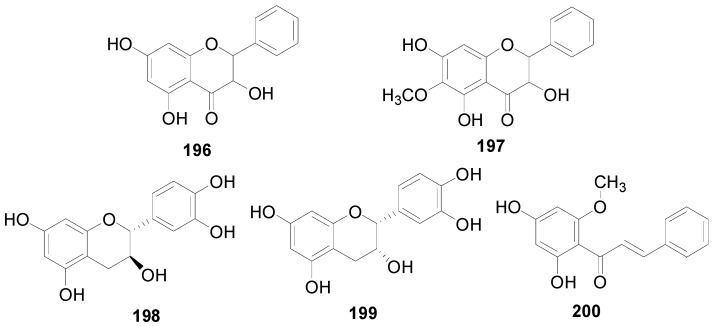
Structures of compounds **196**–**200**.

**Figure 9:**
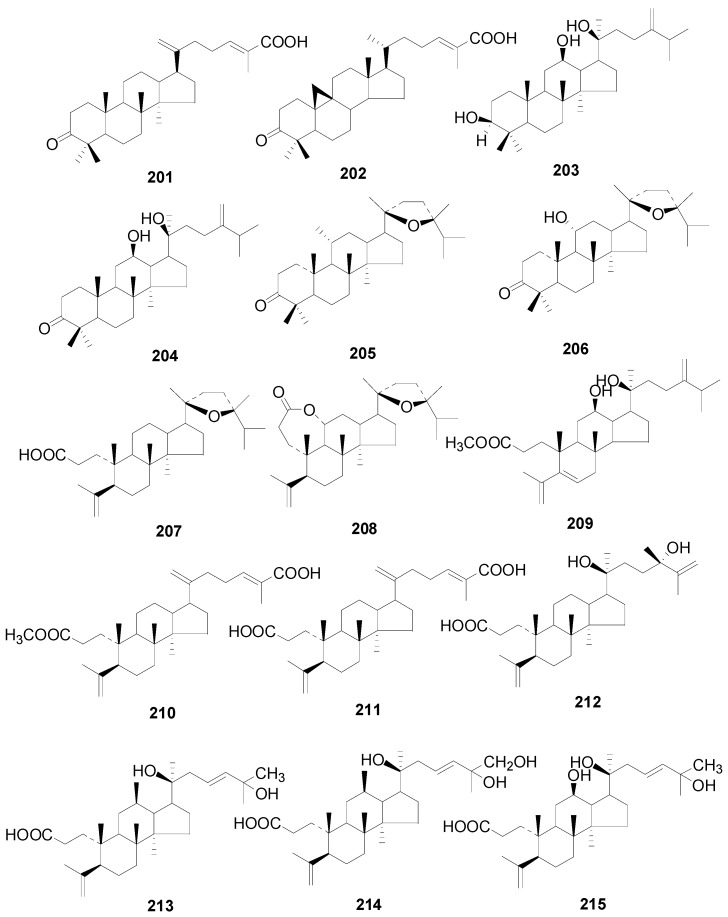
Structures of compounds **201**–**215**.

**Figure 10:**
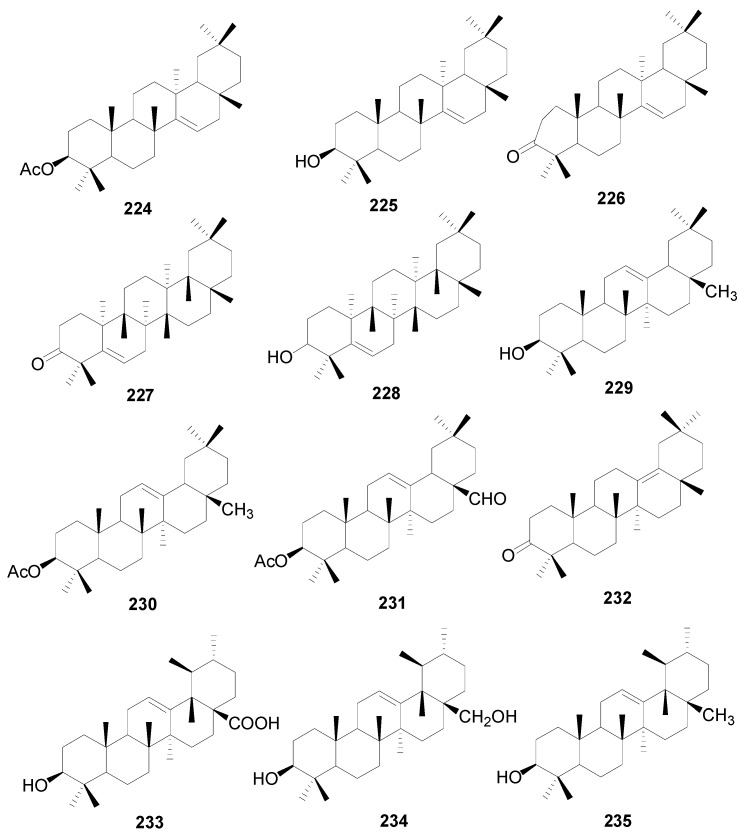
Structures of compounds **224**–**235**.

**Figure 11:**
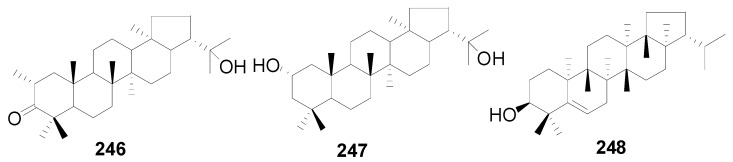
Structures of compounds **246**–**248**.

**Figure 12:**
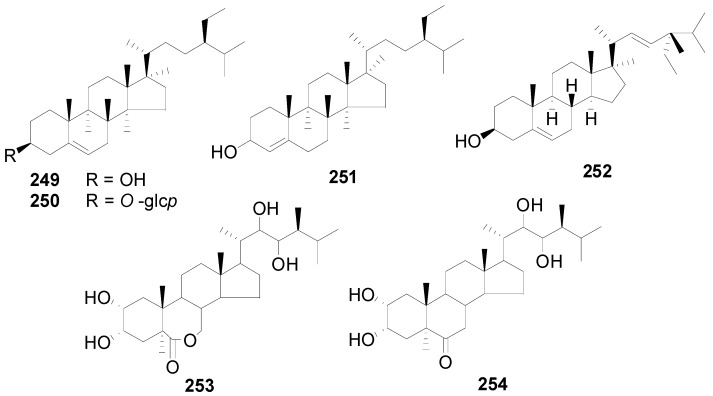
Structures of compounds **249**–**254**.

**Figure 13:**
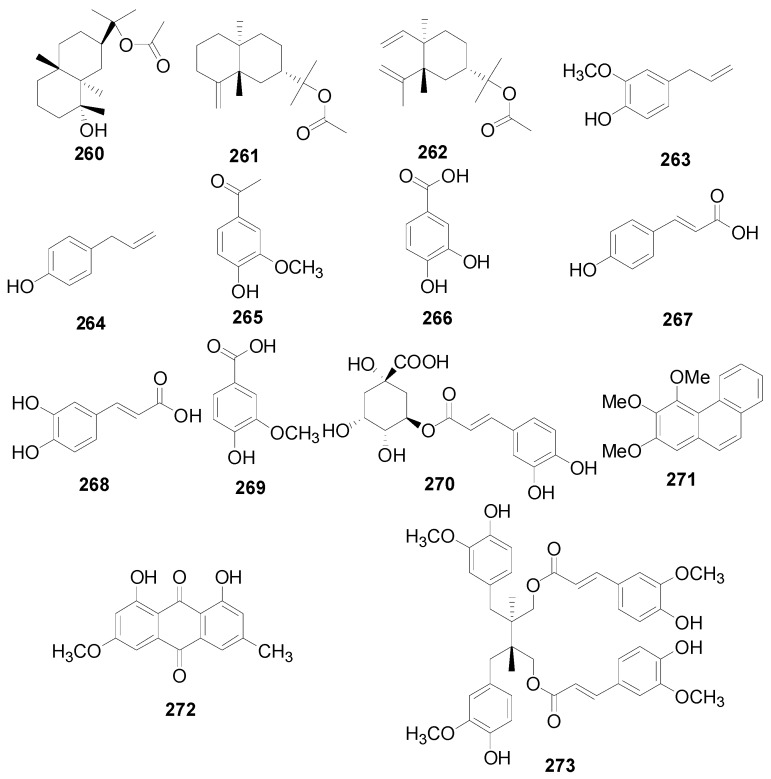
Structures of compounds **260**–**273**.

So far, 273 chemical constituents have been reported from the genus *Alnus*. These compounds can be classified into five groups: diarylheptanoids (compounds **1**–**99**), polyphenols (compounds **100**–**137**), flavonoids (compounds **138**–**200**), terpenoids and steroids (compounds **201**–**254**), and others (compounds **255**–**273**). Their chemical structures are shown in Table 1, Table 2, Table 3, Table 4, Table 5, Table 6, Table 7, Table 8, Table 9, Table 10, Table 11, Table 12, Table 13, Table 14, Table 15 and Table 16 and Figure 1, Figure 2, Figure 3, Figure 4, Figure 5, Figure 6, Figure 7, Figure 8, Figure 9, Figure 10, Figure 11, Figure 12 and Figure 13, and their names and corresponding plant sources are compiled in Table 17. The occurrence of diarylheptanoids appears to be a characteristic feature of this genus.

### 2.1. Diarylheptanoids

The *Alnus* genus has abundant diarylheptanoids containing the 1,7-diphenylheptane frame [21]. Diarylheptanoids have drawn attention due to their physiological activities, especially their anticancer activity [11]. A total of 99 diarylheptanoids have been reported from *Alnus* species. They are categorized into three major groups: linear-type (compounds **1**–**89**), cyclic diphenyl ether-type (compounds **90**–**93**) and cyclic diphenyl-type (compounds **94**–**99**).

Compounds **1**–**89**, the linear-diarylheptanoids shown in Table 1, Table 2 and Table 3 and Figure 1, can be further divided according to the features of the aliphatic carbons. The heptane chains of compounds **1**–**28** (Table 1) are saturated, and the C-3 or C-5 position is always linked to hydroxyls. Diarylheptanoid derivatives **9**–**28** possess a monosaccharide or a disaccharide at the C-3 or C-5 position of the heptane chain to form *O*-glycosides. Moreover, the C-6 position in the glucosyl unit of **26**–**28** is attached to a cinnamoyl moiety. Compounds **29**–**71** (Table 2) are classified as 1,7-bis-(*p*-hydroxyphenyl)-3-heptanones. Compounds **30**–**37** always contain an oxygen substituent at the C-5 position. The hydroxyl at C-5 of **38**–**44** is replaced by an aliphatic hydrocarbon. Diarylheptanoids **45**–**50**, **62**–**69**, as well as **7**, **9**, **14**, **18**, **33**, **38** all could be isolated from the bark of *A*. *glutinosa*. Structure-activity analysis revealed a high dependence of their cytotoxic action on the presence of a carbonyl group at C-3, substitution of the heptane chain on C-5 and the number of hydroxyl groups in the aromatic rings [11]. What′s more, in **53**–**69**, the sugar groups attached at the C-5 position in the heptane group are connected with aromatic acyl radical moieties. These compounds are a class of natural product called cinnamic acid sugar ester derivatives (CASEDs), with one or several phenylacrylic moieties such as -coumaroyl, -cinnamoyl, -benzoyl and -vanilloyl, linked with the non-anomeric carbon of a glycosyl skeleton part through ester bonds. Several CASEDs are reported in traditional medicine as compounds to calm the nerves and display anti-depression and neuroprotective activity [25,26,27]. Compound **52** has a disaccharide unit with β-d-apiofuranosyl bonded to β-d-glucopyranose. Compound **69** is an *O*-(2-methyl)-butanoyl derivative of oregonin (**48**). In addition, compounds **63**–**69** were all isolated from the bark of *A. glutinosa*, and the difference between them is the configuration of the sugar groups in the heptane chain [11]. In alnuside C (**71**), only isolated from *A*. *japonica*, the C-5 hydroxyl on the heptane chain is replaced by a xylose with a methylbutanoyl moiety. The absolute configuration at C-2 of the MeBu unit hasn′t been determined [28].

Compounds **72**–**77** and **78**–**89** are listed in Table 3 and Figure 1, respectively. There are two double bonds at C-1/C-2, C-4/C-5 or C-6/C-7 and a carbonyl at C-3 of **72**–**84**. Meanwhile, **85** and **86** display two carbonyl groups at C-3 and C-5. Hirsutenone (**73**) exhibits prominent antioxidant, anti-inflammatory and anticancer effects [16,21,29]. Compounds **87** and **88** from *A*. *hirsuta* and *Alnus nepalensis* D. Don., respectively, both possess a 1,5-oxy bridge in the carbon chain [30,31]. Alnus dimer (**89**) consists of two units, including a 1,7-bis(3,4-dihydroxyphenyl)-1-(4-hydroxyphenyl)-5-methoxy-heptane-3-one (unit I) and a 1,7-bis (3,4-dihydroxyphenyl)-3-heptanone (unit II), which are connected through a C-C bond between C-3′ of unit I and C-6′ of unit II. Alnus dimer (**89**) showed potent macrofilaricidal and microfilaricidal activity in vitro [32]. Cyclic diarylheptanoids **90**–**99** are grouped into metaparacyclophanes **90**–**93** (Figure 2) and metametacyclophanes **94**–**99** (Figure 3) according to the position of the phenyl groups connected to each other as well as to the heptane chains. The two phenyl groups of compounds **90**–**93** are connected as a diaryl ether. Among them, **90** and **91** with a big cyclic ring, showed potent hypoxia-inducible factor-1 (HIF-1) and nuclear factor-κB (NF-κB) inhibitory activity [30,33]. Acerogenin L (**92**) and garugamblin-3 (**93**) from the methanol extract of *A*. *japonica*, strongly inhibited human low-density lipoprotein oxidation [34]. Compounds **94**–**99** listed in Figure 3, possess two aryl groups coupled at the *meta*-position to the side chain moieties and two hydroxyl groups at C-4′ and C-4′′ of two benzene rings. It was reported that the cyclic diarylheptanoids alnusonol (**95**), alnusdiol (**96**) and alnusone (**98**) always co-occur with the corresponding acyclic derivatives hannokinin (**36**), (+)-hannokinol (**4**) and platyphyllenone (**74**). Some workers have proposed a clear explanation of the biosynthetic relationship between the two types of compounds [35].

### 2.2. Polyphenols

Tannins are the major components of antioxidant polyphenols in genus *Alnus*, mainly including four groups: gallotannins (compounds **100**–**104**), ellagitannins (compounds **105**–**120**), dimeric ellagitannins (compounds **121**–**124**) and C-glycosidic tannins (compounds **125**–**128**). They are always composed of a galloyl group, a hexahydroxydiphenoyl (HHDP) group and a valoneoyl group with glucose core(s) in which the mode of linkage is different. In addition, some polyphenols and their glycosides are also found in this genus.

Table 4 lists compounds **100**–**111**. **100**–**104** from *A*. *japonica*, *A*. *hirsuta* and *Alnus sieboldiana* Matsum. that belong to the gallotannins, in which the galloyl group is directly attached to the hydroxyl of the glucose moiety through an ester bond [5,36,37]. Compound **104** contains a methylated galloyl group, which is linked to the C-1 of the glucose core. Ellagitannins **105**–**111** are esterified by one or two HHDP group(s) at C-1, C-2 and/or C-3, C-4 in the glucosyl moiety to form sugar aryl ester linkages, meanwhile, galloyl groups are often present, except in **108** and **109**. Glutinoin (**111**), a novel antioxidative ellagitannin with a glutinoic acid dilactone moiety, was isolated from *A*. *glutinosa* cones [38]. The C-1 hydroxyl group of the sugar is coupled with a galloyl group, which is connected to the ellagoyl group via the C-4′ (*para*) hydroxyl group. The compound with a *p*-COC-type junction between two units, corresponding to the ellagoyl-galloyl motif in glutinoin, was named glutinoic acid dilactone and the corresponding group was named glutinoyl [38]. To our knowledge, no similar structure has been annotated so far.

Compounds **112**–**115** in Figure 4 isolated from the cones of *A*. *glutinosa*, the leaves of *A*. *japonica* and *Alnus hirsuta* var. *microphylla* based have structures with a valoneoyl group and its depsidone form at C-4/C-6 of the glucose core [5,36,38]. Compared with 4,6-(*S*)-valoneoyl-d-glucose (**113**), flosin A (**112**), praecoxin A (**114**) and praecoxin D (**115**) all possess a HHDP group at C-2/C-3. Additionally, flosin A (**112**) and praecoxin A (**114**) are two isomeric ellagitannins, differing in the orientation of the 4,6-valoneoyl group. Alnusnins A (**116**) and alnusnins B (117) in Figure 4 contain a tergalloyl group at C-4/C-6 and a HHDP group at C-2/C-3 of the glucose core. Compounds **118** and **119** (Figure 5) from the extracts of fruits and leaves of *A*. *sieboldiana* both have a monolactonized tergalloyl group [37,39]. The A, C benzene rings of the tergalloyl group and the HHDP group in alnusiin (**118**) are linked with C-4/C-6 and C-2/C-3 in glucosyl moiety through ester bonds, respectively, while the A and B rings in tergallin (**119**) are attached to the C-2/C-3 positions. Additionally, tergallin (**119**) has a characteristic ellagic acid moiety symmetrically coupled with two galloyl groups at C-4/C-6 by C-C bonds. Hirsunin (**120**, Figure 6), represents a rare example of a hydrolysable tannin, and consists of diarylheptanoid glycoside (oregonin) and ellagitannin (praecoxin A) moieties. It was isolated from *Alnus hirsuta* var. *microphylla* in 1992 and remains the sole example of an ellagitannin with a diarylheptanoid moiety up to now [36,40].

Compounds **121**–**124** in Table 5 and Table 6 are dimeric ellagitannins consisting of pedunculagin (**109**) and strictinin (**107**) units in which the connection position of **124** is different from the other three [5,36]. Furthermore, 1-desgalloylrugosin F (**121**) and rugosin F (**122**) differ from each other on account of the galloyl group being present at C-1 attached to the HHDP group or not. Alnusjaponins A (**123**) and alnusjaponins B (**124**) occurred in *A*. *japonica* are mutually isomeric ellagitannins [5]. C-glucosidic ellagitannins **125**–**128** (Table 7) contain two HHDP groups hold to the C-2/C-3 and C-4/C-6 positions in an open-chain glucose core, one of which participates in the C-glucosidic linkage forming a phenol-aldehyde coupling. Compounds **126**–**128** with a galloyl group at C-1 of the glucosyl unit were isolated from *A*. *sieboldiana* and *A*. *japonica* [5,37,39]. Besides, stenophyllanin A (**128**), a stachyurin-based congener, is linked with a flavan-3-ol unit through the C-C bond between C-1 of glucose and C-8 of flavan-3-ol. Compounds **129**–**133** belong to phenolic glycosides. The hydroxyl at C-6 of glucose core is substituted by syringoyl, vanilloyl or trimethoxycinnamoyl group [41,42]. Shikimic acid (**134**), 5-*O*-galloyl-(−)-shikimic acid (**135**), gallic acid (**136**) and methyl gallate (**137**) are polyphenols with a low molecular weight.

### 2.3. Flavonoids

There are 63 flavonoids in this genus, mainly including flavones, flavonols, flavonones, flavanonols, flavanols and one chalcone. A total of 16 flavones (**138**–**153**), listed in Table 9, were isolated from eight different *Alnus* species [2,6,43,44,45,46,47,48,49]. The hydroxy is always replaced by -CH_3_, -glc, or -glc-glc groups. Flavonols and their derivatives account for a relatively large proportion of the flavonoids in the *Alnus* genus. Flavonols (compounds **154**–**163**) in Table 10 have a C-3 free OH moiety. Compounds **164**–**172** (Table 11) have similar structures with a -OMe function at C-3. Table 12 shows chemical constituents **173**–**188**, in which the C-3 hydroxy is substituted by one or two sugar unit(s). Flavonones **189**–**195** (Table 13) were found in *A. sieboldiana*, *Alnus pendula* Matsum., *Alnus maximowiczii* Call., *Alnus firma* S.Z. and *A. glutinosa* [49,50,51,52,53]. Two flavanonols **197** and **198** are shown in Figure 8. (+)-Catechin (**198**) and (−)-epicatechin (**199**) in Figure 8 are a couple of stereoisomers. Compound **200** (Figure 8) isolated from buds of *Alnus viridis* DC., is the only chalcone among the components reported in the genus *Alnus* [54].

### 2.4. Triterpenoids and Steroids

A total of 48 triterpenes (compound **201**–**248**) shown in Figure 9, Figure 10 and Figure 11 and Table 14 and Table 15 and six steroids (compounds **249**–**254**) in Figure 12 were obtained from the genus *Alnus*. Most of triterpenes are isolated from flowers, leaves and barks of *Alnus* plants [8,30,55,56,57]. They can be assigned into two classes: tetracyclic triterpenes (compounds **201**–**223**) and pentacyclic triterpenes (compounds **224**–**248**).

Triterpenic acids **201** and **202** were both isolated from the leaves of *A*. *nepalensis* [8,31]. Particularly, the cycloartane type mangiferonic acid (**202**), which was not detected in any other *Alnus* species, has been considered as a specific chemical marker of *A. nepalensis* from a chemotaxonomical point of view [8]. The tetracyclic triterpenes **203**–**208** and **216**–**223**, isolated from flowers and leaves of *A*. *sieboldiana*, *Alnus serrulatoides* Call. and *A*. *pendula*, are characterized by their C_31_-dammarane-type and C_31_-3, 4-*seco*-dammarane-type skeletons [55]. All the tetracyclic triterpenes **210**–**215** occurring in *A*. *japonica* male flowers were of the C_30_-3, 4-*seco*-dammarane-type [55]. In particular, alnuselide (**208**), structurally similar to alnuseric acid (**207**), has a lactone ring formed by the 3-carboxyl and the 11-hydroxyl groups [58]. Triterpenoid saponins **218**–**223** are connected with a sugar moiety at C-12 through an *O*-glycosidic bond. In addition, positions C-2 in the sugar unit of compounds **221**–**223** were always linked with an acetyl group.

The isolated pentacyclic triterpenes range from oleananes (compounds **224**–**232**), ursanes (compounds **233**–**235**), lupanes (compounds **236**–**245**), and hopanes (compounds **246**–**247**) to fernanes (**248**). Taraxerol (**225**) is widely spread in *Alnus* species, including *A*. *japonica*, *A*. *hirsuta*, *A*. *nepalensis*, *Alnus maximowiczii* Call., *Alnus acuminata* ssp. *arguta* (Schlecht.) and *Alnus rubra* Bong. [2,8,30,57,59]. Betulinic acid (**239**), the lupane-type triterpenoid acid from the methanol extract of *A*. *hisuta*, potently inhibited rat liver diacylglycerol acyltransferase enzyme activity and triglyceride synthesis in human HepG2 cells [60]. Lup-20(29)en-2,28-diol-3-yl caffeate (**245**) is a novel component with a caffeoyl group at the C-3 position that shows therapeutic potential against liver fibrosis [61].

Moreover, six steroids (compounds **249**–**254**) were obtained from the bark of *A*. *nepalensis* and *A*. *japonica*, the pollen and bark of *A*. *glutinosa*, the stem bark of *A*. *acuminata*, and the aerial part of *A*. *rugosa* L. [48,57,62,63,64].

### 2.5. Other Compounds

About 19 other compounds were isolated from this genus. Five stilbenes **255**–**259** (Table 16) were obtained from the leaves and flowers of *A. sieboldiana*, *A. pendula*, *A. maximowiczii* and *A. viridis* [49,50,53,65]. Compounds **260**–**262** (Figure 13) linked with a carboxyl group are sesquiterpenoid acetates. Compounds **263**–**271** (Figure 13) are low molecular weight phenols. The phenanthrene derivative 2,3,4-trimethoxyphenanthrene (**271**), anthraquinone physcion (**272**), and phenylpropanoid secoisolariciresinol diferulate (**273**) were found in *A. maximowiczii*, *A. nepalensis* and *A. japonica* [8,53,66]. Additionally, physcion (**272**), mangiferonic acid (**202**) together with 22-hydroxyhopan-3-one (**246**) are specific chemical markers of *A. nepalensis* since their distribution has not been detected in any other *Alnus* species [8].

## 3. Biological Activities

Many species of the genus *Alnus* have been used as remedies for diarrhea, dysentery, fever and inflammatory diseases in traditional Chinese and Korean medicine [107]. *Alnus* species have remarkable anticancer activity, which is the most notable and important pharmacological activity [21]. Beyond that, they also display antioxidant, anti-inflammatory, antimicrobial, antiviral and hepatoprotective activities, etc. [16,52,57,105]. Diarylheptanoids are the the main effective components in the genus *Alnus* for their remarkable biological activities [2]. Phenolic compounds and flavonoids, which are widely found as secondary metabolites in *Alnus* plants, are important due to their ability to act as antioxidants [18]. Additionally, several triterpenoids have revealed antitumor activity, HIV-1 viral enzyme inhibition and hepatoprotective effects [52,61,104].

### 3.1. Anticancer Activity

Many researchers have focused on the pharmacological effects of hirsutenone (**73**) isolated from *A*. *japonica*, *A*. *hirsuta* and *A*. *pendula*, etc. [13,69,87]. It has a similar chemical structure and anti-cancer properties as curcumin, the most well-known diarylheptanoid in turmeric (*Curcuma longa* Linn, Zingiberaceae), which has been ranked as a third generation chemoprevention agent by the U.S. National Cancer Institute [21,108,109]. Hirsutenone (**73**) inhibited the tumor promoter 12-*O*-tetradecanoylphorbol-13-acetate (TPA)-induced upregulation of cyclooxygenase-2 (COX-2) and matrix metalloproteinases-9 (MMP-9) in human breast epithelial cells, which has been implicated in the pathogenesis of different kinds of cancer [110]. Further experiments confirmed the cytotoxic activity of **73** against HT-29 human colon carcinoma cells via the induction of oxidative stress and topo II-mediated DNA damage [21]. It can enhance the apoptotic effect of tumor necrosis factor-related apoptosis inducing ligand (TRAIL) on epithelial ovarian carcinoma cell lines by increasing the activation of the caspase-8- and Bid-dependent pathways and mitochondrial pathway, leading to caspase activation as well [111]. Kang et al. found that hirsutenone suppressed human prostate cancer by targeting Akt1 and 2 as a key component to explain for anti-cancer activity [112]. A research in 2014 indicated that hirsutenone (**73**) could sensitize chemoresistant ovarian cancer cells to cisplatin via modulation of apoptosis-inducing factor and X-linked inhibitor of apoptosis [24].

Compounds **46**, **47**, **50**, **62**, **66** and **73** from *A*. *glutinosa* bark exhibited strong anticancer activity compared with other diarylheptanoids from the same species and considerably higher than curcumin, which served as a positive control, in human non-small cell lung carcinoma cell lines. Structure-activity analysis revealed a high dependence of the cytotoxic action on the presence of a carbonyl group at C-3, substitution of a heptane chain on C-5 and the number of hydroxyl groups in the aromatic rings [11]. In addition, Choi et al. found that platyphylloside (**50**), which has a keto-enol moiety and one hydroxyl group in the aromatic ring, showed the most potent cytotoxic activity on B16 mouse melanoma and human stomachic adenocarcinoma cells. This study also suggested that the ketone or keto-enol moiety and hydroxyl groups in the aromatic rings were essential to the higher cytotoxic activity of diarylheptanoids [73].

A rencent comparative study was performed on structurally analogous diarylheptanoids isolated from the bark of *A*. *viridis* and *A*. *glutinosa* to address their biological effects and determine structure-activity relationship. (5*S*)-*O*-methylplatyphyllonol (**40**) and platyphyllenone (**74**) do not possess 3′ and 3′′-OH groups showed significantly higher cytotoxicity compared to that of analogues 5(*S*)-*O*-methylhirsutanonol (**38**) and hirsutenone (**73**). The C-4/C-5 double bond instead of a methoxy group in compounds **74** and **73** positively influenced cell growth inhibition and pro-apoptotic potential. These results indicated that minor differences in the chemical structure can greatly influence the effect of diarylheptanoids on apoptosis and redox status and determine their selectivity towards cancer cells [76].

Oregonin (**48**) and hirsutanonol (**33**) are potential cancer chemopreventive agents. They showed significant inhibitory effects on TPA-induced COX-2 expression in immortalized human breast epithelial MCF10A cells [113]. In addtion, a novel immunomodulator **48** exhibited powerful anticancer activity through augmenting the acttivities of macrophage and natural killer cells [114,115]. Jin et al. isolated six diarylheptanoids from the stem bark of *A*. *hirsuta*. Among them, cyclic-type diarylheptanoids **90** and **91** inhibited the HIF-1 activation with IC_50_ values of 11.2 μM and 12.3 μM while the other diarylheptanoids showed very weak activity with IC_50_ values greater than 100 μM. It suggested that the big cyclic ring contributes to the strong HIF-1 activity [30]. The leaves, barks, and cones extracts of *A*. *incana* and *A*. *viridis* showed potent cytotoxic inhibition effect on HeLa cells with IC_50_ values ranging from 26.02 to 68.5 μg/mL. The most active extract of *A*. *incana* bark has been found to contain great amounts of total phenolics (316.2 mg of gallic acid (**137**)) [116]. Pedunculagin (**109**), which is an ellagitannin, exhibited dose-dependent cytotoxicity in vitro and a lengthening effect on the lifespan in mice bearing S_180_ tumors in vivo [117]. Galangin (**154**), isolated from *A*. *sieboldiana*, significantly inhibited tumor necrosis factor-α (TNF-α) gene expression in A549 cells. It may also be useful in cancer prevention [100].

### 3.2. Antioxidant Activity

Free radical damage is linked to the occurrence of many degenerative diseases, including cancer, cardiovascular disease, cataracts, and aging. Antioxidants can attenuate the damaging effects of reactive oxygen species (ROS) in vitro and have attracted major interest, not only for health care and cosmetics, but also in the food industry [118].

There are many reports that the extracts and isolated compounds from this genus have significant antioxidative activitiy. It has been reported that oregonin (**48**) and hirsutenone (**73**) showed prominent ability to scavenge oxygen radicals compared with the positive controls in the ABTS·^+^ (2,20-azino-bis(3-ethylbenzo-thiazoline)-6-sulphonic acid diammonium salt)-scavenging, superoxide anion radical O_2_^−^, and DPPH (1,1-diphenyl-2-picrylhydrazyl) tests, the total oxidant scavenging capacity (TOSC) assay and the oxygen radical absorbance capacity (ORAC) assay [16,74,90]. It is worth mentioning that compounds **48** and **9** showed stronger antioxidative activities than the well-known antioxidant curcumin [90]. Furthermore, many other compounds also exhibited remarkable free-radical-scavenging capacity; e.g., diarylheptanoids **4**, **14**, **36**, **49**, polyphenols **109**, **111**, **115**, **134** and flavones **144**, **157** [15,38,98,102]. As we all know, polyphenols are natural antioxidants, and the protection against oxidative damage is due to their antioxidant effects. Extracts of *A. incana* and *A. viridis* leaves, bark, and cones were found to be strong DPPH free radical scavengers with IC_50_ values ranging from 3.3 to 18.9 μg/mL. However, correlation with total phenol and tannin contents was not observed. The results showed that the antioxidant effect might be attributed to the presence of other compositions, such as diarylheptanoids and triterpenoids [116]. The ethanolic extracts of *A. nitida* barks and *A. glutinosa* stem barks both also possess the radical scavenging capacity [98,119]. Gallic acid (**136**), rutin (**178**) and (+)-catechin (**198**) are the active constituents responsible for the antioxidant activity of *A. nitida* bark ethanolic extract [119]. Furthermore, the antioxidant properties displayed by the extract of *A. glutinosa* is linked to a successful reduction in inflammatory processes, and the antioxidant potential of *A. nitida* bark might protect from liver damage [22,119].

Structurally, hirsutenone (**73**), hirsutanonol (**33**), oregonin (**48**), rubranoside B (**12**), and rubranoside C (**22**), which possess two 3,4-dihydroxyphenyl rings, were more active against ROS than alnuside A (**45**), and alnuside B (**46**), which have a 3,4-dihydroxyphenyl ring and a 4-hydroxyphenyl ring. Platyphyllone-5-xylose (**47**), platyphyllone (**36**), and platyphylloside (**50**), which have two 4-hydroxyphenyl rings, showed weak activity [16]. From that, we can see that the scavenging capacity against peroxyl radicals is closely related to the phenolic hydroxyls. Some other studies also revealed that the phenolic hydroxyls were essential to the higher antioxidative activity of diarylheptanoids [10,28]. Recently, the combined theoretical and experimental studies confirmed that the catechol moiety as an H-atom donor was very important for the free radical scavenging effect. Thermodynamic descriptors mainly O–H bond dissociation enthalpies (BDEs) establish a clear structure–activity relationship [90]. Oregonin (**48**) and hirsutenone (**73**), together with two cyclic diarylheptanoids, acerogenin L (**92**) and garugamblin-3 (**93**), exhibited significant human LDL-antioxidant activities in the thiobarbituric acid-reactive substance (TBARS) assays with IC_50_ values of 3.2, 1.5, 2.9, 1.7 μM, respectively [34,120].

### 3.3. Anti-Inflammatory Activities

Diarylheptanoids and phenolic glycosides isolated from *A. japonica*, *A. hirsuta*, *A. firma*, *A. formosana*, *A. nitida*, *A. nepalensis* and *A. acuminata* showed significant anti-inflammatory effect [33,41,57,70,99,121,122].

Kim et al. isolated nine known diarylheptanoids from the barks of *A. japonica.* Among these diarylheptanoids, oregonin (**48**) and hirsutenone (**73**) exhibited apparent inhibitory effects on lipopolysaccharide (LPS)-induced NO production and COX-2 production [121]. An analysis of the structure-activity relationship suggested that the presence of a keto-enol group in the heptane moiety or a caffeoyl group in the aromatic ring was important for the inhibitory activity efficacy [121]. Later on, another study also suggested that the carbonyl group is important for the inhibitory activity of diarylheptanoids against LPS-induced NO production [41]. Lee et al. provided new evidence for the anti-inflammatory actions of oregonin (**48**), which include the inhibition of inducible nitric oxide synthase (iNOS) gene transcription via suppressing transcriptional activity of NF-κB and activator protein-1 (AP-1), as well as the up regulation of anti-inflammatory molecule HO-1 [123]. In addition, oregonin (**48**) reduces lipid accumulation, inflammation and ROS production in primary human macrophages, indicating its anti-inflammatory bioactivity [124]. Hirsutenone (**73**) may exert a preventive effect against microbial endotoxin lipopolysaccharide-induced inflammatory skin diseases through inhibition of extracellular signal-regulated kinase (ERK) pathway-mediated NF-κB activation [29,125].

Compounds **12**, **33**, **90** and **91** in the bark of *A. hirsuta*, four phenolic glycosides **129**–**132** and six diarylheptanoids **36**, **41**, **45**, **48**, **50**, **53** isolated from *A. firma* showed significant ability to inhibit LPS-induced inflammation in macrophages or BV2 microglial cells [10,33,41]. Aguilar et al. confirmed the traditional uses of *A. acuminata* in acute inflammatory conditions and its safety for consumption. Several triterpenoids from the hexane extract and diarylheptanoids from the methanol extract of *A. acuminata* were isolated and characterized [57]. The methanol extract of *A. glutinosa* leaves and shikimic acid (**134**) were found to exhibit remarkable anti-inflammatory effect by “inhibition of acetic acid-induced capillary permeability”, “carrageenan-induced hind paw edema” and “TPA-induced ear edema” assays [98]. A recent study in 2017 evaluated the methanol extract and derived fractions of *A. nitida* stem bark for anti-inflammatory activity by using in vitro heat induced albumin denaturation assay and various in vivo assays. It suggested that the presence of polyphenols, sterols, terpenoids and other constituents of *A. nitida* stem bark might contribute towards the anti-inflammatory and analgesic activities [99].

### 3.4. Antimicrobial and Antiviral Activities

It was reported that the EtOH extract of *A. pendula* bark had significant antibacterial activity against methicillin-resistant *Staphylococcus aureus* (MRSA). Oregonin (**48**) and hirsutenone (**73**) isolated from the active fractions inhibited MRSA strains with the minimum inhibitory concentrations (MICs) ranged from 31.25 to 250 μg/mL. Moreover, two fold MIC of **73** could completely suppress the growth of MRSA [126]. Later on, the antibacterial evaluation of the fractions by bioautography on *Staphylococcus aureus* revealed that **48** was the most active, with an antibacterial inhibitory effect comparable to antibiotics [17]. The extracts of cone, leaves, and bark of *A. incana* and *A. viridis* showed antimicrobial activities against 15 microorganisms with MIC values ranging from 0.117 to 0.292 mg/mL [116]. Genkwanin (**143**) isolated from the seeds of *A. glutinosa* showed high antimicrobial activity against seven strains of Gram-positive and Gram-negative bacteria [47]. Betulin (**237**), betulone (**238**) and betulinic acid (**240**) were identified as the major antimycobacterial constituents in the bark of *A. incana*. The functionality at C-3 and C-28 of the lupane skeleton was seemed to be important in determining the antimycobacterial activity [105].

Triterpenoids and flavonoids isolated from *A. firma* were found to inhibit HIV-1 virus replication and controlled its essential enzymes. Alnustic acid methyl ester (**209**) exhibited significant inhibitory effect to HIV-1 protease with IC_50_ value of 15.8 μM, and flavonoids **157**, **176**, **185** inhibited HIV-1 reverse transcriptase all with IC_50_ of 60 μM [52]. Hirsutenone (**73**) showed remarkbable inhibitory effect to papain-like protease with IC_50_ value of 4.1 μM. Furthermore, the authors elucidated that catechol and α,β-unsaturated carbonyl moiety may be responsible for the inhibitory activity [23]. Platyphyllenone (**74**) and platyphyllonol-5-*O*-β-d-xylopyranoside (**47**) showed high anti-viral activity against Influenza A virus H_9_N_2_ with EC_50_ values of 29.9 and 56.1 μM, compared with the positive control, zanamivir (EC_50_ = 16.9 μg/mL), respectively [83]. Betulinic aldehyde (**241**) exhibited anti-influenza effect against KBNP-0028 (H9N2) avian influenza virus with an EC_50_ value of 12.5 μg/mL, compared to a positive control, oseltamivir (EC_50_ = 0.063 μg/mL) [12].

### 3.5. Hepatoprotective Activity

The methanolic extract of the bark of *A. firma* exhibited significant antifibrotic activity. Meawhlie, compounds **73** and **245** isolated from *A. firma* barks showed potent inhibitory effect on the proliferation of hepatic stellate cell (HSC). The authors determined that the presence of substitution at C-3 and C-5 might be responsible for the inhibitory activity of diarylheptanoids on HSC proliferation [61]. It was reported that *A. japonica* was used for hepatitis as an endemic species in Korea. There were evidences that the methanol extract of *A. japonica* stem bark displays hepatoprotective effects against acetaminophen-induced cytotoxicity in cultured rat hepatocytes in vitro [127]. Compounds **12**, **14**, **22**, **33**, **37**, **38**, **45**, **46**, **48**, **49** and **73** isolated from the *A. japonica* bark and the *A. hisuta* stem bark showed significant hepatoprotective effects on tert-butyl hydroperoxide (*t*-BHP) -induced damage to HepG2 cells. According to structure characteristics, the authors considered that the cytoprotective effect was closely related to catechol moiety [3,16,80]. Sajid et al. explored the antioxidant and hepatoprotective properties of *A. nitida* stem bark crude methanol extract on rats. The study concluded that the hepatoprotective activity of *A. nitida* bark is likely due to the antioxidant potential [119]. Furthermore, some other studies also suggested that the hepatoprotective effect is closely linked with the antioxidant property [16,127].

### 3.6. DNA Damage Protection Activity

Novaković et al. isolated twenty-one diarylheptanoids and two polyphenols from the barks of *A. glutinosa* and *A. viridis*. All isolated compositions were evaluated for their in vitro protective effects on chromosome aberrations in peripheral human lymphocytes using cytokinesis-block micronucleus (CBMN) assay. Many of them exerted a pronounced effect of decreasing DNA damage of human lymphocytes, acting stronger than the known synthetic protector amifostine [4,42]. Both platyphylloside (**50**) and **62** isolated from the bark of *A. glutinosa* could protect HaCaT cells against doxorubicin-induced DNA damage. They showed chemo-protective effects of at multiple subcellular levels, and could be considered as protective agents for non-cancerous dividing cells during chemotherapy [19,20].

### 3.7. Anti-Adipogenic Activity

Martineau et al. found that the extracts of the inner bark of *A. incana* strongly inhibited the formation of triglyceride-laden mature adipocytes from 3T3-L1 pre-adipocytes. *A. incana* extracts acted early in the differentiation process and acted as partial agonists toward peroxisome proliferator activated receptor gamma (PPAR-γ) activity. The diarylheptanoid glycoside oregonin (**48**) was isolated and confirmed to be the active principle exerting the anti-adipogenic effect in *A. incana* [82,128]. On the evaluation of antiadipogenic activities of diarylheptanoids isolated from *A. hirsuta*, eighteen compounds were obtained and most of them could decrease lipid accumulation in 3T3-L1 pre-adipocytes. In the assay system, the most potent compound **47**, platyphyllonol-5-*O*-β-d-xylopyranoside had anti-adipogenic activity mediated by the regulation of PPAR-γ dependent pathway. Furthermore, the ketone functionality at C-3, the substituent at C-5, the double bond in heptanone and the hydroxyl groups in the benzene rings were related to the activity [69]. The lupane-type triterpenoid **239** isolated from the methanol extract of *A. hirsuta* showed significant inhibitory activity to diacylglycerol acyltransferase with IC_50_ value of 9.6 μM in the rat liver microsomes. In addition, it also inhibited the triglyceride formation in human HepG2 cells. The results indicated that betulinic acid (**239**) may be a potential lead agent in the treatment of obesity [60].

### 3.8. Anti-Atopic Activity

Atopic dermatitis (AD) is a common inflammatory skin disease. Choi and his co-workers did many studies about the anti-atopic dermatitis effect of oregonin (**48**) and hirsutenone (**73**). The AD animal models were treated with them via topical application as well as intraperitoneal injection. The Th2-related cytokines IL-4, IL-5, IL-13 levels, IgE inflammatory factors, eosinophil levels in blood and lymphocytes were all reduced in AD-like skin lesions of rat model. These results revealed that the two diarylheptanoids were effective for the treatment of AD [129,130]. The leaves and barks extract from *A. japonica* was also proved useful in the treatment of atopic dermatitis and other allergic skin diseases in NC/Nga mice [131]. In addition, hirsutenone is an attractive source for developing a topical drug for T cell-based antiatopic dermatitis by its actions as a calcineurin inhibitor [132]. Furthermore, they developed hirsutenone-loaded and oregonin-loaded Tat peptide-admixed elastic liposomal formulations to treat AD, which aid to increasing the skin permeation of medicine [133,134].

### 3.9. Insecticidal Activity

Research performed by Tung et al. found that the crude extract of *A. japonica* bark showed significant inhibition effect on the growth of *Trypanosoma brucei*. Oregonin (**48**) and hirsutenone (**73**) displayed obvious inhibitory activities against *T. brucei* growth in the bloodstream with IC_50_ of 1.14 and 1.78 μM, respectively. Analysis of their structure–activity relationships revealed that the 3-oxo function of the heptane chain in the diarylheptanoid molecules is necessary for their trypanocidal activity [75]. Compounds **33**, **73**, **74** and **77** isolated from *A. nepalensis* exhibited potential antifilarial activity both in vitro and in vivo studies [135]. Alnus dimer (**89**) and compounds **34** found in *A. nepalensis* showed potent microfilaricidal (LC_100_ = 31.25~62.5 μg/mL, IC_50_ = 11.05~22.10 μg/mL) and macrofilaricidal activities in vitro (LC_100_ = 15.63 μg/mL, IC_50_ = 6.57~10.31 μg/mL) [32].

### 3.10. Other Activities

Apart from the summarized functions above, the constituents or extracts from Alnus plants also have some other activities. Oregonin (**48**) could improve glucose metabolism and insulin signal transduction of HepG2 cells partly by enhancing the expression level of insulin receptor and insulin receptor substrate-1 [85]. The cyclic diarylheptanoids **95**, **98** and **99** isolated from the branch wood of *A. sieboldiana* were assayed for α-glucosidase inhibitory activities with the IC_50_ values 2.34, 8.69 and 1.35 μg/mL, respectively. In comparison, they have a stronger inhibitory effect than acarbose (IC_50_ = 451 μg/mL), a positive control, which is currently used as an antidiabetic agent [97]. Diarylheptanoids **7**, **14**, **33** and **48** isolated from the bark of *A. hirsuta* could reduce melanin level and tyrosinase activity in melanoma cell [136]. Furthermore, it was reported that 5(R)-*O*-methylhirsutanonol (**41**) and oregonin (**48**) might be useful in the prevention and treatment of atherosclerosis through attenuation of adhesion molecule expression by inhibition of NF-κB activation [81]. Hirsutenone (**73**) was able to protect retinal ganglion cells from oxidative stress-induced death. Therefore, it was considered to be a neuroprotective agent for the treatment of neurodegenerative disease, such as glaucoma [137]. The methanol extract of *A. glutinosa* subsp. glutinosa leaves increased wound tension, contraction capacity and tissue hydroxyproline levels. Shikimic acid (**134**) was found to be the major compound responsible for the wound healing effect [88]. The methanolic extract of *A. rugosa* stems showed anticholinesterase activity and it was very interesting for further isolation of acetylcholinesterase inhibitors, which are widely used in the treatment of Alzheimer′s disease [138].

## 4. Conclusions

This article summarized a total of 273 compounds that have been reported from the genus *Alnus*, with 138 cited references. Many species in the genus *Alnus* have been used as traditional herbal medicines in Korea, Japan and China [2]. So far, phytochemical research on the genus has revealed the extensive presence of diarylheptanoids, flavones, polyphenols, terpenoids, steroids and other compound types. The pharmacological activities of pure compounds and crude extract from this genus were mainly focused on anticancer, antioxidant and anti-inflammatory properties. For their significant anticancer activities, diarylheptanoids are a research hotspot in the genus *Alnus*.

Some researchers have pointed out the anticancer mechanism of hirsutenone (**73**), which should be more thoroughly tested as a potential anticancer agent in the future. Some experiments involved the structure-function relationships of antioxidant, anticancer, anti-adipogenic and insecticidal activities, but were not deep enough except structure-antioxidant activity relationship [11,69,75,90]. Hence, researchers may need to pay attention to them in future studies. As a whole, the phytochemical and biological investigations were mainly concentrated on the seven *Alnus* species (*A*. *japonica*, *A*. *hirsuta*, *A*. *glutinosa*, *A*. *incana*, *A*. *nepalensis*, *A*. *sieboldiana* and *A*. *firma*), with little or no attention being paid to other folk medical species. In view of this background, plenty of further studies are necessary in order to examine the other plants of the *Alnus* genus, extend the use of *Alnus* species and provide clinical rationale for the development of new therapeutic agents from traditional medicine sources. The authors hope this review will provide valuable data for the exploration and advanced research on *Alnus* species.

## Figures and Tables

**Table 1:**
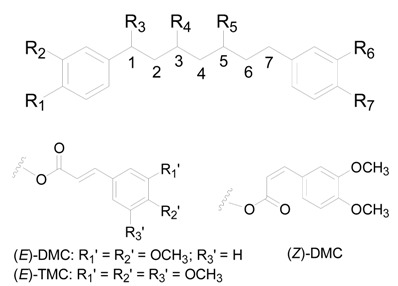
Structures of compounds **1**–**28**.

Compound	R_1_	R_2_	R_3_	R_4_	R_5_	R_6_	R_7_
**1**	H	H	H	OH (*R*)	OH (*S*)	H	H
**2**	H	H	H	OH (*R*)	OH (*R*)	H	H
**3**	H	H	OH (*R*)	OH (*R*)	OH (*S*)	H	H
**4**	OH	H	H	OH (*R*)	OH (*R*)	H	OH
**5**	OH	H	H	OH (*R*)	H	H	OH
**6**	OH	H	H	OH (*R*)	H	OH	OH
**7**	OH	OH	H	OH (*R*)	H	OH	OH
**8**	OH	OH	H	OH (*R*)	H	H	OH
**9**	OH	OH	H	OH (*R*)	*O*-xyl*p* (*S*)	OH	OH
**10**	OH	H	H	OH (*R*)	*O*-api*f*(1→6)glc*p*	H	OH
**11**	OH	H	H	*O*-xyl*p* (*R*)	H	H	OH
**12**	OH	OH	H	*O*-xyl*p* (*R*)	H	OH	OH
**13**	OH	OH	H	*O*-xyl*p* (*R*)	H	H	OH
**14**	OH	OH	H	*O*-glc*p* (*R*)	H	OH	OH
**15**	OH	H	H	*O*-glc*p* (*R*)	H	OH	OH
**16**	OH	OH	H	*O*-glc*p* (*R*)	H	H	OH
**17**	OH	H	H	*O*-glc*p* (*R*)	OH	H	OH
**18**	OH	H	H	*O*-glc*p* (*R*)	H	H	OH
**19**	OH	H	H	*O*-api*f*(1→6)glc*p* (*R*)	H	H	OH
**20**	OH	H	H	*O*-ara*f*(1→6)glc*p* (*S*)	H	H	OH
**21**	OH	H	H	*O*-ara*f*(1→6)glc*p* (*R*)	H	H	OH
**22**	OH	OH	H	*O*-glc*p*(1→3)xyl*p* (*R*)	H	OH	OH
**23**	OH	OH	H	*O*-api*p*(1→6)glc*p* (*R*)	H	H	OH
**24**	OH	OH	H	*O*-rha*p*(1→6)glc*p* (*R*)	H	H	OH
**25**	OH	H	H	*O*-glc*p*(1→3)xyl*p* (*R*)	H	H	OH
**26**	OH	OH	H	*O*-glc*p*-(*E*)-DMC (*R*)	H	OH	OH
**27**	OH	OH	H	*O*-glc*p*-(*Z*)-DMC (*R*)	H	OH	OH
**28**	OH	OH	H	*O*-glc*p*-(*E*)-TMC (*R*)	H	OH	OH

**Table 2:**
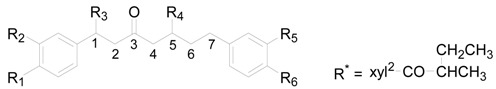
Structures of compounds **29**–**71**.

Compound	R_1_	R_2_	R_3_	R_4_	R_5_	R_6_
**29**	OH	H	H	H	H	OH
**30**	H	H	OH (*S*)	OH (*S*)	H	H
**31**	H	H	OH (*R*)	OH (*S*)	H	H
**32**	H	H	H	OH (*S*)	H	H
**33**	OH	OH	H	OH (*S*)	OH	OH
**34**	OH	OH	H	OH (*S*)	H	OH
**35**	OH	H	H	OH (*S*)	OH	OH
**36**	OH	H	H	OH (*S*)	H	OH
**37**	OH	OH	H	OH (*R*)	OH	OH
**38**	OH	OH	H	OCH_3_ (*S*)	OH	OH
**39**	OH	H	H	OCH_3_ (*S*)	OH	OH
**40**	OH	H	H	OCH_3_ (*S*)	H	OH
**41**	OH	OH	H	OCH_3_ (*R*)	OH	OH
**42**	OH	OH	H	*O*-*^n^*Bu (*S*)	OH	OH
**43**	OH	OH	H	*O*-*^n^*Bu (*S*)	OH	OH, △^1^(E)
**44**	OH	H	H	*O*-*^n^*Bu (*S*)	H	OH
**45**	OH	H	H	*O*-xyl (*S*)	OH	OH
**46**	OH	OH	H	*O*-xyl (*S*)	H	OH
**47**	OH	H	H	*O*-xyl (*S*)	H	OH
**48**	OH	OH	H	*O*-xyl (*S*)	OH	OH
**49**	OH	OH	H	*O*-glc (*S*)	OH	OH
**50**	OH	H	H	*O*-glc (*S*)	H	OH
**51**	OH	H	H	*O*-glc (*S*)	OH	OH
**52**	OH	OH	H	*O*-glc (*S*)	H	OH
**53**	OH	H	H	*O*-api*f*(1→6)glc*p* (*S*)	H	OH
**54**	OH	H	H	*O*-galloyl-glc*p* (*S*)	H	OH
**55**	OH	OH	H	*O*-xyl*p*-*p*-coumaroyl	OH	OH
**56**	OH	OH	H	*O*-xyl*p*-feruloyl (*S*)	OH	OH
**57**	OH	OH	H	*O*-galloyl-glc*p* (*S*)	OH	OH
**58**	OH	OH	H	*O*-xyl*p*-benzoyl (*S*)	OH	OH
**59**	OH	OH	H	*O*-xyl*p*-cinnamoyl (*S*)	OH	OH
**60**	OH	OH	H	*O*-glc*p*-benzoyl (*S*)	OH	OH
**61**	OH	OH	H	*O*-glc*p*-vanilloyl (*S*)	OH	OH
**62**	OH	H	H	*O*-glc*p*-coumaroyl (*S*)	H	OH
**63**	OH	H	H	*O*-glc*p*-(*E*)-DMC (*S*)	H	OH
**64**	OH	H	H	*O*-glc*p*-(*E*)-DMC (*S*)	OH	OH
**65**	OH	H	H	*O*-glc*p*-(*Z*)-DMC (*S*)	H	OH
**66**	OH	OH	H	*O*-glc*p*-coumaroyl (*S*)	OH	OH
**67**	OH	OH	H	*O*-glc*p*-(*Z*)-DMC (*S*)	OH	OH
**68**	OH	OH	H	*O*-glc*p*-(*E*)-TMC (*S*)	OH	OH
**69**	OH	OH	H	*O*-glc*p*-(*E*)-DMC (*S*)	OH	OH
**70**	OH	OH	H	*O*-xyl*p*-2-methyl-butanoyl (*S*)	OH	OH
**71**	OH	OH	H	*O*-R* (*S*)	OH	OH

**Table 3:**
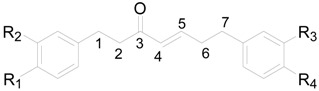
Structures of compounds **72**–**77**.

Compound	R_1_	R_2_	R_3_	R_4_
**72**	H	H	H	H
**73**	OH	OH	OH	OH
**74**	OH	H	H	OH
**75**	OH	H	OCH_3_	OH
**76**	OH	OH	H	OH
**77**	OH	H	OH	OH

**Table 4:**
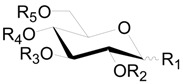
Structures of compounds **100**–**111**.

Compound	R_1_	R_2_	R_3_	R_4_	R_5_
**100**	OH	H	H	G	G
**101**	OG	H	H	G	H
**102**	OG	H	H	G	H
**103**	OG	G	H	H	H
**104**	A	H	H	H	H
**105**	OH	H	G	(*S*) HHDP	
**106**	OH	G	G	(*S*) HHDP	
**107**	OG	H	H	(*S*) HHDP	
**108**	OH	(*S*) HHDP		H	H
**109**	OH	(*S*) HHDP		(*S*) HHDP	
**110**	β-OG	(*S*) HHDP		(*S*) HHDP	
**111**	OB	(*S*) HHDP		(*S*) HHDP	

**Table 5:**
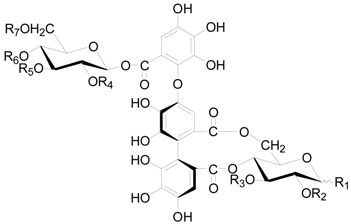
Structures of compounds **121**–**123**.

Compound	R_1_	R_2_	R_3_	R_4_	R_5_	R_6_	R_7_
**121**	OH	(*S*) HHDP		G	G	(*S*) HHDP	
**122**	β-OG	(*S*) HHDP		G	G	(*S*) HHDP	
**123**	OH	(*S*) HHDP		H	H	(*S*) HHDP	

**Table 6:**
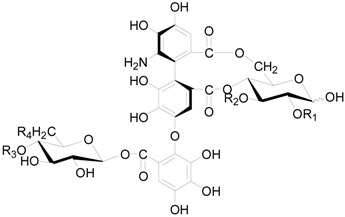
Structures of compounds **124**.

Compound	R_1_	R_2_	R_3_	R_4_
**124**	(*S*) HHDP		(*S*) HHDP	

**Table 7:**
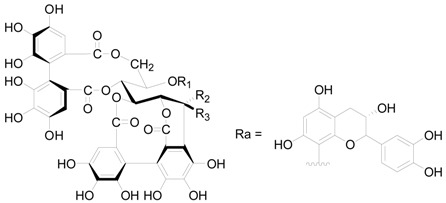
Structures of compounds **125**–**128**.

Compound	R_1_	R_2_	R_3_
**125**	H	H	OH
**126**	G	H	OH
**127**	G	OH	H
**128**	G	Ra	H

**Table 8:**
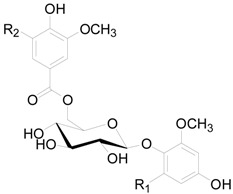
Structures of compounds **129**–**132**.

Compound	R_1_	R_2_
**129**	OCH_3_	OCH_3_
**130**	OCH_3_	H
**131**	H	OCH_3_
**132**	H	H

**Table 9:**
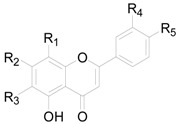
Structures of compounds **138**–**153**.

Compound	R_1_	R_2_	R_3_	R_4_	R_5_
**138**	H	OH	H	H	H
**149**	H	OH	H	H	OH
**140**	H	OH	H	OH	OCH_3_
**141**	H	OH	H	H	OCH_3_
**142**	H	OCH_3_	H	H	H
**143**	H	OCH_3_	H	H	OH
**144**	H	OCH_3_	H	OH	OCH_3_
**145**	H	OCH_3_	H	OCH_3_	OH
**146**	OCH_3_	OCH_3_	OCH_3_	H	H
**147**	H	OCH_3_	OCH_3_	H	OCH_3_
**148**	H	*O*-glc*p*-glc*p*	H	H	OH
**149**	H	OH	H	H	*O*-glc*p*-glc*p*
**150**	H	OCH_3_	H	H	OCH_3_
**151**	H	OH	OCH_3_	H	OCH_3_
**152**	H	OH	H	OH	OH
**153**	H	*O*-glc	H	OH	OH

**Table 10:**
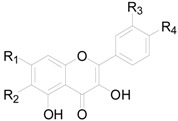
Structures of compounds **154**–**163**.

Compound	R_1_	R_2_	R_3_	R_4_
**154**	OH	H	H	H
**155**	OH	H	H	OH
**156**	OH	H	H	OCH_3_
**157**	OH	H	OH	OH
**158**	OH	H	OCH_3_	OH
**159**	OH	OCH_3_	H	H
**160**	OH	OCH_3_	H	OCH_3_
**161**	OCH_3_	H	H	H
**162**	OCH_3_	H	OH	OH
**163**	OCH_3_	H	OCH_3_	OCH_3_

**Table 11:**
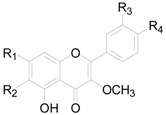
Structures of compounds **164**–**172**.

Compound	R_1_	R_2_	R_3_	R_4_
**164**	OH	H	H	H
**165**	OH	H	OCH_3_	OH
**166**	OH	OCH_3_	H	OH
**167**	OH	OCH_3_	H	OCH_3_
**168**	OH	OCH_3_	OH	OCH_3_
**169**	OCH_3_	H	H	OH
**170**	OCH_3_	H	OH	OH
**171**	OCH_3_	H	OH	OCH_3_
**172**	OCH_3_	OCH_3_	H	H

**Table 12:**
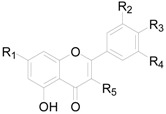
Structures of compounds **173**–**188**.

Compound	R_1_	R_2_	R_3_	R_4_	R_5_
**173**	OH	OH	OH	H	*O*-ara*f*
**174**	OH	OH	OH	H	*O*-glc*p*
**175**	OH	H	OH	OH	*O*-glc*p*
**176**	OH	OH	OH	H	*O*-rha*p*
**177**	OH	OH	OH	H	*O*-glucuronide
**178**	OH	OH	OH	H	*O*-rha*p*(1→6)glc*p*
**179**	OH	OH	OH	H	*O*-cel
**180**	OH	OH	OH	H	*O*-mal
**181**	OH	H	OH	H	*O*-rha
**182**	OH	OH	OH	H	*O*-gal*f*
**183**	OH	H	OH	H	*O*-rha-rha
**184**	OH	OH	OH	H	*O*-sop
**185**	OH	OH	OH	OH	*O*-gal*p*
**186**	OCH_3_	OH	OH	H	*O*-glc*p*-glc*p*
**187**	OH	OCH_3_	OH	H	*O*-glc
**188**	*O*-rha	OCH_3_	OH	H	*O*-glc

**Table 13:**
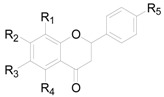
Structures of compounds **189**–**195**.

Compound	R_1_	R_2_	R_3_	R_4_	R_5_
**189**	H	OH	H	OH	H
**190**	H	OH	H	OH	OH
**191**	H	OH	H	OCH_3_	H
**192**	H	OH	CH_3_	OH	H
**193**	H	OCH_3_	H	OH	H
**194**	H	OH	OH	OH	OH
**195**	CH_3_	OH	CH_3_	OH	OH

**Table 14:**
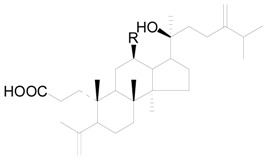
Structures of compounds **216**–**223**.

Compound	R
**216**	H
**217**	OH
**218**	*O*-xyl*p*
**219**	*O*-glc*p*
**220**	*O*-ara*p*
**221**	*O*-(2′-OAc)-ara*f*
**222**	*O*-(2′-OAc)-xyl*p*
**223**	*O*-(2′-OAc)-glc*p*

**Table 15:**
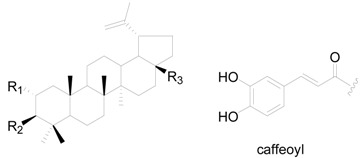
Structures of compounds **236**–**245**.

Compound	R_1_	R_2_	R_3_
**236**	H	OH	CH_3_
**237**	H	OH	CH_2_OH
**238**	H	O	CH_2_OH
**239**	H	OH	COOH
**240**	H	OH	OH
**241**	H	OH	CHO
**242**	H	OCOCH_3_	CHO
**243**	H	OCOCH_3_	CH_3_
**244**	H	O	CH_3_
**245**	OH	*O*-caffeoyl	CH_2_OH

**Table 16:**
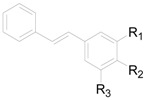
Structures of compounds **255**–**259**.

Compound	R_1_	R_2_	R_3_
**255**	OH	H	OH
**256**	OH	H	OCH_3_
**257**	OCH_3_	OH	OH
**258**	H	H	H
**259**	OCH_3_	H	OCH_3_

**Table 17:** Chemical Constituents from the Genus *Alnus*.

No.	Compound Class and Name	Source	Reference
	*Diarylheptanoids*		
**1**	yashabushidiol A	*A*. *sieboldiana*, *Alnus fruticosa* Rupr., *Alnus mandshurica* (Callier) Hand.-Mazz	[2,67,68]
**2**	yashabushidiol B	*A*. *sieboldiana*, *A*. *fruticosa*, *A*. *mandshurica*	[2,67,68]
**3**	yashabushitriol	*A*. *sieboldiana*	[2,68]
**4**	(+)-hannokinol	*A*. *hirsuta*, *A*. *japonica*	[35,69]
**5**	(−)-centrolobol	*Alnus formosana* Burk., *A*. *nepalensis*, *A*. *acuminata*, *A*. *hirsuta*	[31,57,70,71]
**6**	(±)-7-(3,4-dihydroxyphenyl)-1-(4-hydroxyphenyl)-3-heptanol	*A*. *formosana*	[70]
**7**	rubranol	*A*. *hirsuta*, *A*. *japonica*, *A*. *rubra*, *A*. *formosana*	[2,23,69,70,72]
**8**	1-(3,4-dihydroxyphenyl)-7-(4-hydroxyphenyl)-3 (*R*)-heptanol	*A*. *formosana*	[70]
**9**	(3*R*,5*S*)-1,7-bis-(3,4-dihydroxyphenyl)-3-hydroxylheptane-5-*O*-β-d-xylopyranoside	*A*. *japonica*, *A*. *glutinosa*, *A*. *incana*	[11,73,74]
**10**	5-hydroxy-1,7-bis(4-hydroxyphenyl)heptan-3-yl β-d-apiofuranosyl-(1→6)-β-d-glucopyranoside	*A*. *viridis*	[42]
**11**	1,7-di(4-hydroxyphenyl)-3(*R*)-β-d-xylosyloxyheptane.	*A*. *formosana*	[70]
**12**	rubranoside B	*A*. *hirsuta*, *A*. *rubra*, *A*. *japonica*, *A*. *formosana*, *A*. *glutinosa*	[2,4,69,70]
**13**	alnuside C	*A*. *japonica*	[75]
**14**	rubranoside A	*A*. *hirsuta*, *A*. *japonica*, *A*. *rubra*, *A*. *incana*, *A*. *formosana*, *A*. *glutinosa*	[2,3,4,70,73,74]
**15**	7-(3,4-dihydroxyphenyl)-1-(4-hydroxyphenyl)-3(*R*)-β-d-glucosyloxyheptane	*A*. *formosana*	[70]
**16**	1-(3,4-dihydroxyphenyl)-7-(4-hydroxyphenyl)-3(*R*)-β-d-glucosyloxyheptane	*A*. *formosana*, *A*. *japonica*	[70,75]
**17**	(1*S*,3*R*)-3-hydroxy-5-(4-hydroxyphenyl)-1-[2-(4-hydroxyphenyl)ethyl]pentyl β-d-glucopyranoside	*A*. *viridis*	[76]
**18**	aceroside VII	*A*. *hirsuta*, *A*. *formosana*, *A*. *glutinosa*, *A*. *viridis*	[2,3,4,42,70]
**19**	aceroside VIII	*A*. *hirsuta*, *A*. *viridis*	[42,69]
**20**	(1*S*)-5-(4-hydroxyphenyl)-1-[2-(4-hydroxyphenyl)ethyl]pentyl 6-*O*-α-L-arabinofuranosyl-β-d-glucopyranoside	*A*. *viridis*	[76]
**21**	(3*R*)-1,7-bis(4-hydroxyphenyl)heptan-3-yl *α*-L-arabinofuranosyl-(1→6)-β-d-glucopyranoside	*A*. *viridis*	[42]
**22**	rubranoside C	*A*. *japonica*, *A*. *hirsuta*, *A*. *rubra*	[2,3,73]
**23**	rubranoside D	*A*. *japonica*. *A*. *rubra*	[2,73]
**24**	alnuside D	*A*. *japonica*	[75]
**25**	(3*R*)-1,7-bis-(4-dihydroxyphenyl)-3-heptanol-3-*O*-β-d-glucopyranosyl(1→3)-β-d-xylopyranoside	*A*. *hirsuta*	[2,3]
**26**	3(*R*)-1,7-di(3,4-dihydroxyphenyl)-3-*O*-β-d-[6-(*E*-3,4-dimethoxycinnamoyl glucopyranosyl)] heptane	*A*. *glutinosa*	[11]
**27**	3(*R*)-1,7-di(3,4-dihydroxyphenyl)-5-*O*-β-d-[6-(*Z*-3,4-dimethoxycinnamoyl glucopyranosyl)] heptane	*A*. *glutinosa*	[11]
**28**	3(*R*)-1,7-di(3,4-dihydroxyphenyl)-5-*O*-β-d-[6-(*E*-3,4,5-trimethoxycinnamoyl glucopyranosyl)] heptane	*A*. *glutinosa*	[11]
**29**	1,7-bis-(*p*-hydroxyphenyl)-3-heptanone	*A*. *nepalensis*	[11]
**30**	yashabushiketodiol B	*A*. *sieboldiana*	[2,68]
**31**	yashabushiketodiol A	*A*. *sieboldiana*	[2,68]
**32**	dihydroyashabushiketol	*A*. *firma*, *A*. *sieboldiana*, *A*. *maximowiczii*	[2,54]
**33**	hirsutanonol	*A*. *hirsuta*, *A*. *japonica*, *A*. *rubra*, *A*. *glutinosa*, *A*. *formosana*, *A*. *acuminata*, *A*. *serrulatoides*	[11,13,57,70,77,78,79]
**34**	5(*S*)-1-(3,4-dihydroxyphenyl)-7-(4-hydroxyphenyl)-5-hydroxyheptane-3-one	*A*. *japonica*	[80]
**35**	5(*S*)-1-(4-dihydroxyphenyl )-7-(3,4-dihydroxyphenyl)-5-hydroxyheptane-3-one	*A*. *japonica*, *A*. *nepalensis*, *A*. *hirsuta*	[32,69,80]
**36**	hannokinin	*A*. *japonica*, *A*. *nepalensis*, *A*. *hirsuta*, *A*. *firma*	[16,32,41,69]
**37**	epihirsutanonol	*A*. *japonica*	[80]
**38**	5(*S*)-*O*-methylhirsutanonol	*A*. *japonica*, *A*. *glutinosa*, *A*. *formosana*, *A*. *nepalensis*	[11,32,70,81]
**39**	alunheptanoid A	*A*. *japonica*	[63]
**40**	5(*S*)-*O*-methylplatyphyllonol	*A*. *japonica*	[63]
**41**	5(*R*)-*O*-methylhirsutanonol	*A*. *japonica*	[63]
**42**	5-*O*-butylhirusutanonol	*A*. *formosana*	[70]
**43**	5(*S*)-butyloxy-1,7-di(3,4-dihydroxyphenyl)-1(*E*)-hepten-3-one	*A*. *formosana*	[70]
**44**	5(*S*)-butyloxy-1,7-di(4-hydroxyphenyl)-3-heptanone	*A*. *formosana*	[70]
**45**	alnuside A	*A*. *japonica*, *A*. *serrulatoides*, *A*. *hirsuta*, *A*. *formosana*, *A*. *glutinosa*, *A*. *incana*	[2,11,28,70,79,82]
**46**	alnuside B	*A*. *japonica*, *A*. *serrulatoides*, *A*. *hirsuta*, *A*. *formosana*, *A*. *glutinosa*, *A*. *incana*	[2,11,28,70,79,82]
**47**	platyphyllonol-5-*O*-β-d-xylopyranoside	*A*. *rubra*, *A*. *hirsuta*, *A*. *japonica*, *A*. *glutinosa*	[11,69,83,84]
**48**	oregonin	*A*. *japonica*, *A*. *hirsuta*, *A*. *rubra*, *A*. *nepalensis*, *A*. *glutinosa*, *A*. *firma*, *A*. *formosana*, *A*. *incana*, *A*. *serrulatoides*, *A*. *pendula*, *A*. *tinctoria* Sarg.	[2,4,41,70,74,79,85,86,87,88,89]
**49**	5(*S*)-hirsutanonol-5-*O*-β-d-glucopyranoside	*A*. *hirsuta*, *A*. *japonica*, *A*. *rubra*, *A*. *incana*, *A*. *formosana*, *A*. *serrulatoides*, *A*. *acuminata*, *A*. *nepalensis*, *A*. *glutinosa*	[2,3,11,57,70,74,79,84,86,88]
**50**	platyphylloside	*A*. *japonica*, *A*. *hirsuta*, *A*. *glutinosa*, *A*. *formosana*, *A*. *pendula*, *A*. *firma*, *A*. *incana*, *A*. *nepalensis*, *A*. *rubra*, *A*. *viridis*	[2,3,11,24,41,73,84,87,88,90,91]
**51**	(5*S*)-1-(4-hydroxyphenyl)-7-(3,4-dihydroxy-phenyl)-5-*O*-β-d-glucopyranosyl-heptan-3-one	*A*. *glutinosa*	[4]
**52**	1-(3′,4′-dihydroxypheny1)-7-(4′′-hydroxypheny1)-5-*O*-β-d-glucopyranosylheptan-3-one	*A*. *rubra*	[72]
**53**	(5*S*)-5-hydroxy-1,7-bis-(4-hydroxyphenyl)-3-heptanone-5-*O*-β-d-apiofuranosyl-(1→6)-β-d-glucopyranoside	*A*. *hirsuta*, *A*. *viridis*	[42,69]
**54**	(3*S*)-1,7-bis(4-hydroxyphenyl)-5-oxoheptan-3-yl 6-*O*-galloyl-β-d-glucopyranoside	*A*. *viridis*	[42]
**55**	oregonoyl A	*A*. *japonica*, *A*. *formosana*	[2,70,83]
**56**	oregonoyl B	*A*. *japonica*	[2,83]
**57**	hirsutanonol 5-*O*-(6-*O*-galloyl)-β-d-glucopyranoside	*A*. *japonica*	[2,92]
**58**	2′′′-*O*-benzoyl-oregonin	*A*. *formosana*	[70]
**59**	2′′′-*O*-cinnamoyl-oregonin	*A*. *formosana*	[70]
**60**	oregonoside A	*A*. *rubra*	[78]
**61**	oregonoside B	*A*. *rubra*	[78]
**62**	5(*S*)-1,7-di(4-hydroxyphenyl)-5-*O*-β-d-[6-(*E*-*p*-coumaroyl glucopyranosyl)]heptane-3-one	*A*. *glutinosa*	[11]
**63**	5(*S*)-1,7-di(4-hydroxyphenyl)-5-*O*-β-d-[6-(*E*-3,4-dimethoxycinnamoyl glucopyranosyl)]heptane-3-one	*A*. *glutinosa*	[11]
**64**	5(*S*)-1-(4-hydroxyphenyl)-7-(3,4-dihydroxyphenyl)-5-*O*-β-d-[6-(*E*-3,4-dimethoxycinnamoyl glucopyranosyl)]heptane-3-one	*A*. *glutinosa*	[11]
**65**	5(*S*)-1-(3,4-dihydroxyphenyl)-7-(4-hydroxyphenyl)-5-*O*-β-d-[6-(*Z*-3,4-dimethoxycinnamoyl glucopyranosyl)]heptane-3-one	*A*. *glutinosa*	[11]
**66**	5(*S*)-1,7-di(3,4-dihydroxyphenyl)-5-*O*-β-d-[6-(*E*-*p*-coumaroyl glucopyranosyl)]heptane-3-one	*A*. *glutinosa*	[11]
**67**	5(*S*)-1,7-di(3,4-dihydroxyphenyl)-5-*O*-β-d-[6-(*Z*-3,4-dimethoxycinnamoyl glucopyranosyl)] heptane-3-one	*A*. *glutinosa*	[11]
**68**	5(*S*)-1,7-di(3,4-dihydroxyphenyl)-5-*O*-β-d-[6-(*E*-3,4,5-trimethoxycinnamoyl glucopyranosyl)] heptane-3-one	*A*. *glutinosa*	[11]
**69**	5(*S*)-1,7-di(3,4-dihydroxyphenyl)-5-*O*-β-d-[6-(*E*-3,4-dimethoxycinnamoyl glucopyranosyl)] heptane-3-one	*A*. *glutinosa*	[11]
**70**	2′′′-*O*-(2-methylbutanoyl)-oregonin	*A*. *formosana*	[70]
**71**	1,7-bis-(3,4-dihydroxyphenyl)-5-hydroxy-3-heptanone-5-*O*-[2-(2-methylbutenoyl)]-β-d-xylopyranoside	*A*. *japonica*	[2,28]
**72**	1,7-diphenylhept-3-en-5-one	*A. maximowiczii*	[2]
**73**	hirsutenone	*A*. *japonica*, *A*. *hirsuta*, *A*. *pendula*, *A*. *nepalensis*, *A*. *glutinosa*, *A*. *firma*, *A*. *formosana*, *A*. *acuminata*	[2,8,11,28,41,57,69,70,87]
**74**	platyphyllenone	*A*. *hirsuta*, *A*. *japonica*, *A*. *formosana*, *A*. *rubra A*. *acuminata*, *A*. *viridis*	[2,3,16,42,57,70,93]
**75**	1-(4-hydroxyphenyl)-7-(4-hydroxy-3-methoxyphenyl)-4-hepten-3-one	*A*. *hirsuta*	[2,30]
**76**	1-(3′,4′-dihydroxyphenyl)-7-(4′′-hydroxyphenyl)-4-hepten-3-one	*A*. *japonica*, *A*. *rubra*	[2,16,93]
**77**	alusenone	*A*. *japonica*	[2,13]
**78**	nitidone A	*Alnus nitida* Endl.	[94]
**79**	nitidone B	*Alnus nitida* Endl.	[94]
**80**	yashabushiketol	*A*. *firma*, *A*. *sieboldiana*, *A*. *hirsuta*	[2,71,95,96]
**81**	(5*S*)-hydroxy-1-(3,4-dihydroxyphenyl)-7-(4-hydroxyphenyl)-hepta-1*E*-en-3-one	*A*. *hirsuta*	[69]
**82**	alnustone	*A*. *pendula*, *A*. *japonica*	[2,35]
**83**	1,7-bis-(3,4-dihydroxyphenyl)-hepta-4*E*,6*E*-dien-3-one	*A*. *hirsuta*	[69]
**84**	1,4-hepta-dien-3-one-1,7-bis(3,4-dihydroxyphenyl)-(1*E*,4*E*)	*A*. *hirsuta*	[69]
**85**	1,7-diphenylheptane-3,5-dione	*A*. *maximowiczii*	[2,53]
**86**	1,7-diphenylhept-1-ene-3,5-dione	*A*. *maximowiczii*	[2,53]
**87**	rhoiptelol B	*A*. *hirsuta*	[2,30]
**88**	1,5-epoxy-1-(3′,4′-dihydroxyphenyl)-7-(4′′-hydroxyphenyl)heptane	*A*. *nepalensis*	[31]
**89**	alnus dimer	*A*. *nepalensis*	[32]
**90**	*trans*-rhoiptelol	*A*. *hirsuta*	[2,9,30]
**91**	myricatomentogenin	*A*. *hirsuta*	[2,9,30]
**92**	acerogenin L	*A*. *japonica*	[2,34]
**93**	garugamblin-3	*A*. *japonica*	[2,34]
**94**	alnusoxide	*A*. *japonica*	[35]
**95**	alnusonol	*A*. *japonica*, *A*. *hirsuta*, *A*. *sieboldiana*	[35,71,97]
**96**	alnusdiol	*A*. *japonica*, *A*. *hirsuta*	[35,71]
**97**	trideoxysasadanin-8-ene	*A*. *hirsuta*	[71]
**98**	alnusone	*A*. *japonica*, *A*. *hirsuta*, *A*. *sieboldiana*	[35,71,90,97]
**99**	3,17-dihydroxy-tricyclo[12.3.1.1 ^2,6^]-nonadeca-1(18),2,4,6(19),14, 16-hexaen-9,11-dione	*A*. *sieboldiana*	[97]
	*Polyphenols*		
**100**	4,6-di-*O*-galloyl-d-glucose	*A*. *japonica*	[2,5]
**101**	1,4-di-*O*-galloyl-β-d-glucose	*A*. *japonica*	[2,5]
**102**	1,4,6-tri-*O*-galloyl-β-d-glucose	*A*. *hirsuta*	[2,36]
**103**	1,2,6-tri-*O*-galloyl-β-d-glucose	*A*. *hirsuta*, *A*. *sieboldiana*	[2,36,37]
**104**	gentisic acid 5-*O*-β-d-(6′-*O*-galloyl) glucopyranoside	*A*. *hirsuta*	[2,36]
**105**	gemin D	*A*. *japonica*	[2,5]
**106**	tellimagrandin I	*A*. *hirsuta*, *A*. *sieboldiana*	[2,36,37]
**107**	strictinin	*A*. *japonica*, *A*. *sieboldiana*	[5,37]
**108**	2,3-*O*-(*S*)-hexahydroxydiphenoyl-d-glucose	*A*. *japonica*, *A*. *sieboldiana*	[2,5]
**109**	pedunculagin	*A*. *japonica*, *A*. *sieboldiana*, *A*. *hirsuta*, *A*. *glutinosa*	[2,5,36,38,39]
**110**	1(β)-*O*-galloylpendunculagin	*A*. *japonica*, *A*. *sieboldiana*	[2,37]
**111**	glutinoin	*A*. *glutinosa*	[38]
**112**	flosin A	*A*. *japonica*	[2,5]
**113**	4,6-(*S*)-valoneoyl-d-glucose	*A*. *japonica*	[2,5]
**114**	praecoxin A	*A*. *japonica*, *A*. *hirsuta*	[2,5,36]
**115**	praecoxin D	*A*. *glutinosa*	[38]
**116**	alnusnins A	*A*. *sieboldiana*	[2,37]
**117**	alnusnins B	*A*. *sieboldiana*	[2,37]
**118**	alnusiin	*A*. *sieboldiana*	[2,39]
**119**	tergallin	*A*. *sieboldiana*	[37]
**120**	hirsunin	*A*. *hirsuta*	[2,36]
**121**	1-desgalloylrugosin F	*A*. *hirsuta*	[2,36]
**122**	rugosin F	*A*. *hirsuta*	[2,36]
**123**	alnusjaponins A	*A*. *japonica*	[2,5]
**124**	alnusjaponins B	*A*. *japonica*	[2,5]
**125**	casuariin	*A*. *sieboldiana*	[2,39]
**126**	casuarinin	*A*. *japonica*, *A*. *sieboldiana*	[2,5,39]
**127**	stachyurin	*A*. *japonica*, *A*. *sieboldiana*	[2,5,37]
**128**	stenophyllanin A	*A*. *sieboldiana*	[2,37]
**129**	4-hydroxy-2,6-dimethoxyphenyl-6′-*O*-syringoyl-β-d-glucopyranoside	*A*. *firma*	[2,41]
**130**	4-hydroxy-2,6-dimethoxyphenyl-6′-*O*-vanilloyl-β-d-glucopyranoside	*A*. *firma*	[2,41]
**131**	4-hydroxy-2-methoxyphenyl-6′-*O*-syringoyl-β-d-glucopyranoside	*A*. *firma*	[41]
**132**	6′-*O*-vanilloylisotachioside	*A*. *firma*	[41]
**133**	methyl 3,4-dihydroxy-5-{[6-*O*-(3,4,5-trimethoxycinnamoyl) -β-d-glucopyranosyl]oxy}benzoate	*A*. *viridis*	[42]
**134**	shikimic acid	*A*. *japonica*	[98]
**135**	5-*O*-galloyl-(−)-shikimic acid	*A*. *japonica*	[2,5]
**136**	gallic acid	*A*. *nepalensis*, *A*. *nitida*	[8,99]
**137**	methyl gallate	*A*. *sieboldiana*	[100]
	*Flavonoids*		
**138**	chrysin	*A*. *sieboldiana*	[2,49]
**139**	apigenin	*A*. *rubra*, *A*. *sieboldiana*, *A*. *rugosa*	[2,44,46,48]
**140**	diosmetin	*A*. *rugosa*	[48]
**141**	acacetin	*A*. *japonica*, *A*. *rubra*, *Alnus koehnei* Call.	[2,6,44]
**142**	tectochrysin	*A*. *sieboldiana*	[2,49]
**143**	genkwanin	*A. sinuata*, *A. glutinosa*	[2,6,47]
**144**	5,3′-dihydroxy-7,4′-dimethoxyflavone	*A*. *japonica*	[2,6]
**145**	rhamnazin	*A*. *japonica*	[2,6]
**146**	5-hydroxy-6,7,8-tritmethoxyflavone	*A*. *sieboldiana*	[2,45,49]
**147**	salvigenin	*A*. *japonica*, *A*. *rubra*, *A*. *koehnei*	[2,6,44]
**148**	apigenin 7-β-cellobioside	*A*. *sieboldiana*	[46]
**149**	apigenin 4′-β-cellobioside	*A*. *sieboldiana*	[46]
**150**	5-hydroxy-4′,7-dimethoxyflavone	A. japonica, A. acuminata, A. rubra	[2,6,43,44]
**151**	scutellarein-6,4′-dimethyl ether	*A*. *japonica*, *A*. *rubra*	[2,6,44]
**152**	luteolin	*A*. *rugosa*	[48]
**153**	luteolin 7-*O*-β-glucside	*A. rugosa*	[48]
**154**	galangin	*A*. *sieboldiana*, *A*. *pendula*, *A*. *viridis*	[2,50,54,100]
**155**	kaempferol	*A*. *koehnei*, *A*. *sieboldiana*	[6,46]
**156**	kaempferide	*A*. *japonica*, *A*. *koehnei*	[2,6]
**157**	quercetin	*A*. *japonica*, *A*. *nepalensis*, *A*. *firma*, *A*. *formosana*, *A. sieboldiana*	[2,8,91,100,101,102]
**158**	isorhamnetin	*A*. *japonica*, *A*. *koehnei*	[2,6]
**159**	alnusin	*A*. *sieboldiana*, *A*. *pendula*	[2,49,50]
**160**	the 6,4′-dimethyl ether of 6-hydroxykaempferol	*A*. *koehnei*	[2,6]
**161**	izalpinin	*A*. *sieboldiana*	[2,50]
**162**	rhamnetin	*A*. *koehnei*	[2,6]
**163**	quercetin-7,3′,4′-trimethyl ether	*A*. *japonica*, *A*. *koehnei*	[2,6]
**164**	galangin 3-methyl ether	*A*. *viridis*	[54]
**165**	quercetin-3,3′-dimethyl ether	*A*. *koehnei*	[2,6]
**166**	3,6-dimethyl ether of 6-hydroxykaempferol	*A*. *koehnei*	[2,6]
**167**	3,6,4′-trimethyl ether of 6-hydroxy-kaempferol	*A*. *japonica*, *A*. *koehnei*	[2,6]
**168**	quercetagetin-3,6,4′-trimethyl ether	*A*. *koehnei*	[2,6]
**169**	kumatakenin	*Alnus crispa* Pursh., *Alnus sinuate* Rydbg.	[2,6]
**170**	quercetin 3,7-dimethyl ether	*A*. *crispa*, *A*. *koehnei*, *A*. *sinuata*	[2,6]
**171**	quercetin-3,7,4′-trimethyl ether (ayanin)	*A*. *crispa*	[2,6]
**172**	5-hydroxy-3,6,7-trimethoxyflavone	*A*. *sieboldiana*	[2,49]
**173**	quercetin-3-*O*-α-l-arabinofuranoside	*A*. *firma*	[2,52]
**174**	isoquercitrin	*A*. *firma*	[2,52,53]
**175**	quercetin-3-*O*-glucoside	*A*. *formosana*, *A*. *nepalensis*	[32,91]
**176**	quercitrin	*A*. *firma*, *A. formosana*, *A*. *nepalensis*, *A*. *japonica*	[2,8,28,52,91]
**177**	quercetin-3-*O*-β-d-glucuronide	*A*. *sieboldiana*	[2,37]
**178**	rutin	*A*. *nitida*	[99]
**179**	quercetin-3-β-cellobioside	*A*. *sieboldiana*	[46]
**180**	quercetin-3-β-maltoside	*A*, *sieboldiana*	[46]
**181**	kaempferol 3-*O*-rhamnoside	*A*. *japonica*, *A*. *formosana*	[28,91]
**182**	quercetin-3-*O*-galactoside	*A*. *japonica*, *A*. *nepalensis*	[8,28]
**183**	kaempferol-3-dirhamnoside	*A*. *sieboldiana*	[46]
**184**	quercetin-3-sophoroside	*A*. *gultinosa*, *Alnus cordata* Loisel.	[2,32]
**185**	myricetin-3-*O*-β-d-galactopyranoside	*A*. *firma*	[2]
**186**	rhamnetin-3-*O*-rhamnoside	*A*. *formosana*	[91]
**187**	isorhamnetin 3-*O*-β-glucoside	*A*. *rugosa*	[48]
**188**	isorhamnetin 3-β-*O*-glucoside-7-*O*-α-rhamnoside	*A*. *rugosa*	[48]
**189**	pinocembrin	*A*. *sieboldiana*, *A*. *pendula*, *A*. *maximowiczii*, *A*. *firma*	[50,52,53,100]
**190**	naringenin	*A. sieboldiana*	[2,49]
**191**	alpinetin	*A*. *pendula*, *A*. *firma*, *A*. *sieboldiana*	[2,49,50]
**192**	strobopinin	*A*. *sieboldiana*	[2,49]
**193**	pinostrobin	*A*. *pendula*, *A*. *firma*, *A*. *sieboldiana*	[2,49,50]
**194**	rhododendrin	*A*. *glutinosa*	[2,51]
**195**	pinobanksin	*A*. *sieboldiana*	[2,49]
**196**	alnustinol	*A*. *maximowiczii*, *A*. *firma*, *A*. *sieboldiana*, *A*. *pendula*	[2,49,50,53]
**197**	3,5,8-trihydroxy-7-methoxyflavone	*A*. *sieboldiana*	[45]
**198**	(+)-catechin	*A*. *firma*, *A*. *viridis*	[2,42,52]
**199**	(−)-epicatechin	*A*. *firma*	[2,52]
**200**	2′,4′-dihydroxy-6′-methoxychalcone	*A*. *viridis*	[54]
	*Terpenoids*		
**201**	24-(*E*)-3-oxodammara-20 (21),24-dien-27-oic acid	*A*. *nepalensis*	[32]
**202**	mangiferonic acid	*A*. *nepalensis*	[8]
**203**	alnuserrutriol	*A*. *serrulatoides*	[2,55]
**204**	alnuserrudiolone	*A*. *sieboldiana*, *A*. *serrulatoides*	[2,55,103]
**205**	alnincanone	*A*. *serrulatoides*	[2,55]
**206**	alnuserol	*A*. *serrulatoides*	[2,55]
**207**	alnuseric acid	*A*. *serrulatoides*, *A*. *pendula*	[2,55,58,104]
**208**	alnuselide	*A*. *serrulatoides*	[2,55,58]
**209**	alnustic acid methyl ester	*A*. *firma*	[2,52]
**210**	methyl(24*E*)-3,4-secodammara-4(28),20,24-trien-26-oic acid-3-oate	*A*. *japonica*	[2,55]
**211**	(24*E*)-3,4-secodammara-4 (28),20,24-trien-3,26-dioic acid	*A*. *japonica*	[2,55]
**212**	(20*S*,24*S*)-20,24-dihydroxy-3,4-secodammara-4 (28),25-dien-3-oic acid	*A*. *japonica*	[2,55]
**213**	(23*E*)-(20*S*)-20,25-dihydroxy-3,4-secodammara-4 (28),23-dien-3-oic acid	*A*. *japonica*	[2,55]
**214**	(23*E*)-(20*S*)-20,25,26-trihydroxy-3,4-secodammara-4 (28),23-dien-3-oic acid	*A*. *japonica*	[2,55]
**215**	(23*E*)-(12*R*,20*S*)-12,20,25-trihydroxy-3,4-secodammara-4 (28),23-dien-3-oic acid	*A*. *japonica*	[2,55]
**216**	(20*S*)-20-hydroxy-24-methylene-3,4-secodammar-4 (28)-en-3-oic acid	*A*. *pendula*	[2,55]
**217**	alnustic acid	*A*. *serrulatoides*, *A*. *pendula*, *A*. *sieboldiana*	[2,7,55,103]
**218**	alnustic acid-12-*O*-β-d-xylopyranoside	*A*. *serrulatoides*, *A*. *pendula*, *A*. *sieboldiana*	[2,7,55,103]
**219**	alnustic acid-12-*O*-β-d-glucopyranoside	*A*. *serrulatoides*, *A*. *pendula*, *A*. *sieboldiana*	[2,7,55,103]]
**220**	alnustic acid-12-*O*-α-l-arabinofuranoside	*A*. *serrulatoides*, *A*. *pendula*, *A*. *sieboldiana*	[2,7,55,103]
**221**	alnustic acid-12-*O*-(2′-*O*-acetyl)-α-l-arabinofuranoside	*A*. *serrulatoides*, *A*. *pendula*	[2,7,55]
**222**	alnustic acid-12-*O*-(2′-*O*-acetyl)-β-d-xylopyranoside	*A*. *serrulatoides*, *A*. *pendula*	[2,7,55]
**223**	alnustic acid-12-*O*-(2′-*O*-acetyl)-β-d-glucopyranosid	*A*. *serrulatoides*, *A*. *pendula*	[2,7,55]
**224**	taraxeryl acetate	*A*. *japonica*, *A*. *hirsuta*, *A*. *nepalensis*, *A*. *acuminata*	[2,8,30,57,92]
**225**	taraxerol	*A*. *japonica*, *A*. *hirsuta*, *A*. *nepalensis*, *A*. *maximowiczii*, *A*. *acuminata*, *A*. *rubra*	[2,8,30,57,59]
**226**	taraxerone	*A*. *japonica*, *A*. *rubra*, *A*. *nepalensis*, *A*. *glutinosa*, *A*. *acuminata*	[2,56,57]
**227**	glutenone	*A*. *japonica*, *A*. *rubra*, *A*. *fruticosa*, *A*. *kamtschatica*	[2,72]
**228**	glutinol	*A*. *japonica*	[2,92]
**229**	β-amyrin	*A*. *japonica*, *A*. *fruticosa*, *A*. *kamtschatica*, *A*. *firma*, *A*. *glutinosa*	[2,52,56]
**230**	3-*O*-acetyl-β-amyrin	*A*. *japonica*, *A*. *firma*	[2,52]
**231**	3β-acetoxy-olean-12-ene-28-al	*A*. *acuminata*	[57]
**232**	δ-amyrone	*A*. *acuminata*	[43]
**233**	ursolic acid	*A*. *glutinosa*	[56]
**234**	uvaol	*A*. *glutinosa*	[56]
**235**	*α*-amyrin	*A*. *fruticosa*, *A*. *kamtschatica*	[2]
**236**	lupeol	*A*. *japonica*, *A*. *rubra*, *A*. *nepalensis*, *A*. *glutinosa*, *Alnus oregona* Nutt., *A*. *acuminata*	[2,12,56,57,104]
**237**	betulin	*A*. *hirsuta*, *A*. *rubra*, *A*. *nepalensis*, *A*. *japonica*, *A*. *glutinosa*, *A*. *maximowiczii*, *A*. *oregona*	[2,7,8,12,30,56,59,104]
**238**	betulone	*A. incana*	[105]
**239**	betulinic acid	*A*. *japonica*, *A*. *hirsuta*, *A*. *nepalensis*	[2,8,30,63]
**240**	3β,28-dihydroxy-lup-20(29)-ene	*A*. *acuminata*	[57]
**241**	betulinic aldehyde	*A*. *japonica*, *A*. *glutinosa*, *A*. *acuminata*	[12,56,57]
**242**	3-acetoxybetulinic aldehyde	*A*. *japonica*	[12]
**243**	lupenylacetate	*A*. *glutinosa*	[56]
**244**	lupenone	*A*. *japonica*, *A*. *rubra*, *A*. *fruticosa*, *A*. *kamtschatica*, *A*. *glutinosa*	[2,56]
**245**	lup-20(29)en-2,28-diol-3-yl caffeate	*A*. *firma*	[61]
**246**	22-hydroxyhopan-3-one	*A*. *nepalensis*	[8]
**247**	2-hydroxydiploterol	*A*. *nepalensis*	[8]
**248**	simiarenol	*A*. *glutinosa*	[56]
	*Steroids*		
**249**	β-sitosterol	*A*. *japonica*, *A*. *fruticosa*, *A*. *rubra*, *A*. *nepalensis*, *A*. *kamtschatica*, *A*. *firma*, *A*. *glutinosa*, *A*. *acuminata*, *A*. *rugosa*	[2,8,48,52,57,63,64,106]
**250**	β-sitosterol 3-*O*-β-d-glucopyranoside	*A*. *japonica*, *A*. *nepalensis*, *A*. *acuminata*, *A*. *rugosa*	[2,8,48,57,63,64]
**251**	β-rosasterol	*A*. *nepalensis*	[64]
**252**	stigmasterol	*A*. *nepalensis*	[64]
**253**	brassinolide	*A*. *glutinosa*	[62]
**254**	castasterone	*A*. *glutinosa*	[62]
	*Others*		
**255**	pinosylvin	*A*. *sieboldiana*, *A*. *pendula*	[2,49,50]
**256**	pinosylvin monomethyl ether	*A*. *sieboldiana*, *A*. *pendula*, *A*. *maximowiczii*	[2,49,50,53]
**257**	4′,5′-dihydroxy-3′-methoxy stilbene	*A*. *viridis*	[65]
**258**	trans-stilbene	*A*. *firma*, *A*. *sieboldiana*	[2,49,96]
**259**	pinosylvin dimethyl ether	*A*. *sieboldiana*, *A*. *maximowiczii*	[2,49,53]
**260**	cryptomeridiol 11-*O*-monoacetate	*A*. *maximowiczii*	[2,53]
**261**	β-eudesmol acetate	*A*. *maximowiczii*	[2,53]
**262**	elemol acetate	*A*. *maximowiczii*	[2,53]
**263**	eugenol	*A*. *pendula*	[2,50]
**264**	chavicol	*A*. *pendula*	[2,50]
**265**	vanillin	*A*. *nepalensis*	[64]
**266**	protocatechuic acid	*A*. *firma*, *A*. *formosana*	[91,101]
**267**	*p*-coumaric acid	*A*. *firma*	[101]
**268**	caffeic acid	*A*. *firma*	[101]
**269**	vanilic acid	*A*. *japonica*	[66]
**270**	chlorogenic acid	*A*. *firma*	[101]
**271**	2,3,4-trimethoxyphenanthrene	*A*. *maximowiczii*	[2,53]
**272**	physcion	*A*. *nepalensis*	[8]
**273**	secoisolariciresinol diferulate	*A*. *japonica*	[66]
